# Activation of GABBR1 by New Ligand Valerate Transcriptionally Regulates ATF4-NRF2-CXCL13 Axis Mediating CD8^+^ T Cell Anti-Tumor Immunity

**DOI:** 10.7150/ijbs.127859

**Published:** 2026-04-16

**Authors:** Zhiran Zhai, Jumin Huang, Jiayue Qiu, Yuwei Wang, Jin Liao, Chun Xie, Jiahui Xu, Zebo Jiang, Yajia Xie, Wenkang Ren, Yizhong Zhang, Haoxin Yan, Meifang Wang, Chen Huang, Elaine Lai Han Leung

**Affiliations:** 1 School of Pharmacy, Faculty of Chinese Medicine & State Key Laboratory of Mechanism and Quality of Chinese Medicine, Dr. Neher's Biophysics Laboratory for Innovative Drug Discovery, Macau University of Science and Technology, Taipa, Macau, China.; 2 Cancer Center, Faculty of Health Sciences, Ministry of Education (MOE) Frontiers Science Center for Precision Oncology, University of Macau, Macau, Macau SAR, China.; 3 Zhuhai UM Science & Technology Research Institute, University of Macau, Hengqin, Macau SAR, China.; 4 State Key Laboratory of Mechanism and Quality of Chinese Medicine, University of Macau, Macau.; 5 College of Pharmacy, Shaanxi University of Chinese Medicine, Xianyang, Shaanxi, China.; 6 Department of Respiratory and Critical Care Medicine, Taihe Hospital, Hubei University of Medicine, Shiyan, China.; 7 Hubei Key Laboratory of Embryonic Stem Cell Research, Hubei University of Medicine, Shiyan, China.

**Keywords:** valerate, GABBR1, non-small cell lung cancer, anti-tumor immunity

## Abstract

Lung cancer has the highest mortality rate globally, yet the effective treatment options remain limited. Although short-chain fatty acids (SCFAs) like acetate, propionate, and butyrate are known to modulate immune response and improve the efficacy of cancer therapy, the role of valerate in non-small cell lung cancer (NSCLC) remains unexplored. This study identifies valerate as a biased ligand for GABA_B_ receptor, which specifically binds the GABBR1 subunit, upregulates its expression, and thereby mediates CD8^+^ T cell anti-tumor immunity in NSCLC. It is demonstrated that GABBR1 overexpression in lung cancer cells suppresses tumor growth both *in vitro* and *in vivo*. Single-cell RNA sequencing analysis of public datasets indicates that GABBR1 expression is associated with CXCL13. Mechanically, *in vitro* and *in vivo* experiments validate that valerate-induced upregulation of GABBR1 stimulates the secretion of CXCL13, which in turn promotes robust infiltration and activation of CD8^+^ T cells within the tumor microenvironment. Furthermore, enrichment analysis pinpoints that CXCL13 secretion is negatively transcriptionally regulated by NRF2 and its co-factor ATF4. Analysis of patient samples further confirms that high GABBR1 expression correlates with a longer patient survival rate, underscoring its prognostic value. Overall, these findings identify valerate as a novel immunomodulatory metabolite and propose targeting GABBR1 as a promising strategy to potentiate cancer immunotherapy in lung cancer.

## Introduction

Lung cancer is the most frequently diagnosed cancer globally (12.4% of total cases) and the leading cause of cancer death (18.7%) 1. Non-small cell lung cancer (NSCLC) accounts for approximately 85% of all lung cancer cases 2. Surgical resection is the primary treatment for early-stage NSCLC; however, over 80% of patients are diagnosed at an advanced stage, rendering them ineligible for surgery 3. Alternative treatments, including radiotherapy and chemotherapy, show limited efficacy due to side effects and drug resistance. Furthermore, advanced treatment, including targeted therapies and immune checkpoint blockade (ICB) while beneficial for some patients, is effective in a minority of cases and is frequently complicated by acquired resistance 4-7. Despite significant therapeutic advances over the past decade, NSCLC remains largely incurable for most patients.

Short-chain fatty acids (SCFAs), such as acetate, propionate, butyrate, and valerate, were recently found to be attractive targets for novel, physiologically integrated, and potentially less iatrogenic therapies in cancer prevention and treatment 8. A cohort study of 52 patients with solid tumors revealed that elevated valeric acid and other SCFAs concentrations significantly correlated with prolonged progression-free survival 9. Most of the studies focused on acetate, propionate, and butyrate, however, the anti-tumor effect of valerate, specifically in NSCLC is poorly understood. Preclinically, *L. acidophilus-derived* valeric acid inhibited non-alcoholic fatty liver disease-associated hepatocellular carcinoma (NAFLD-HCC) by suppressing Rho GTPase signaling through GPR41/43 10. Valeric acid also acted as an immune regulator, which directly enhanced anti-tumor immunity by augmenting CD8^+^ T cell responses through increased production of CD25, IFN-γ and TNF-α in melanoma and pancreatic cancer models 11. Moreover, in breast cancer, valeric acid exerted therapeutic effects through epigenetic modifications, including DNA methylation and histone deacetylation (HDAC) 12. In addition, we recently observed increased valeric acid levels following combined ginseng polysaccharides (GPs) and anti-PD-1 antibody treatment in NSCLC 13. However, whether valeric acid specifically suppresses lung tumor progression remains unknown.

Valeric acid has a similar structure to neurotransmitter γ-aminobutyric acid (GABA) but lacks the amine functional group, and has been shown to exert neuroprotective effects in the brain partly through elevating GABA levels 14. Although GABA is best known for its role in neural signaling, its receptors, including the metabotropic GABA_B_ receptor, are also widely expressed in non-neural tissues 15, 16. However, the function of GABA_B_ receptors in non-neural tumors remains relatively unexplored. Emerging evidence suggests that GABA_B_ receptor signaling may play a tumor suppressive role in certain cancers. For instance, recent studies in colorectal cancer demonstrated that the GABA_B_ receptor activation inhibited tumor progression by suppressing epithelial-mesenchymal transition (EMT) and Hippo/YAP pathway 17, 18. Furthermore, GABA_B_ receptor activation could suppress proliferation and promote apoptosis across multiple cancer cell lines 19-21. Yet, the underlying mechanism is poorly characterized, hindering the identification of novel therapeutic targets.

In this study, we demonstrated that valerate augmented CD8^+^ T cell anti-tumor activity by activating GABA_B_ receptor, leading to suppression of lung cancer progression. Mechanically, we identified valerate as a direct ligand for GABBR1 and elucidated a downstream signaling cascade involving ATF4 and NRF2, which subsequently induced CXCL13 expression and enhanced CD8^+^ T cell infiltration into tumors. Collectively, these findings delineate a previously unrecognized immunometabolic axis by valerate in lung cancer and highlight valerate as a promising therapeutic candidate, supporting its potential application in combination immunotherapy strategies.

## Materials and Methods

### Cell Culture and Treatment Compounds

The human NSCLC cell lines NCI-A549 (#CRM-CCL-185, RRID: CVCL_0023), NCI-H358 (#CRL-5807, RRID: CVCL_1559), NCI-H2122 (#CRL-5985, RRID: CVCL_1531), NCI-H23 (#CRL-5800, RRID: CVCL_1547), HCC827 (#CRL-2868, RRID: CVCL_2063), NCI-H1975 (#CRL-5908, RRID: CVCL1511), NCI-H1299 (#CRL-5803, RRID: CVCL_0060), the human large cell lung cancer NCI-H460 (#HTB-177, RRID: CVCL_0459), the human bronchial epithelial cell line BEAS-2B (#CRL-3588, RRID: CVCL_0168), the human embryonic kidney cell line HEK293T (CRL-3519, RRID: CVCL_0063), and the mouse Lewis lung carcinoma (LLC1; #CRL-1642, RRID: CVCL_4358) were purchased from the American Type Culture Collection (ATCC). The human NSCLC cell line PC-9 (#CL-0668, RRID: CVCL_B260) and squamous cell lung cancer cell line NCI-H520 (#CL-0402, RRID: CVCL_1566) were purchased from Procell Life Science & Technology. The LLC cells with luciferase and GFP marker (LLC-Luc-GFP; #STCC00071P) was purchased from Servicebio. All cell lines were tested and confirmed to be free of mycoplasma contamination. Primary CD8^+^T cells were isolated and purified from murine spleen by using CD8a^+^ T Cell Isolation Kit (#130-104-075, Miltenyi Biotec). A549, H358, H2122, H23, HCC827, H1975, H1299, H460 and H520 cells were cultured in RPMI 1640 Medium (#11875119, Gibco) supplemented with 10% of Fetal Bovine Serum (FBS; #10270106, Gibco) and 1% penicillin-streptomycin (100 U/mL; #15140122, Gibco). BEAS-2B, HEK293T, LLC and LLC-Luc-GFP cells were cultured in Dulbecco's Modified Eagle Medium (DMEM; #11965092, Gibco) supplemented with 10% FBS and 1% penicillin-streptomycin. All cell lines were cultured at 37 °C in a humidified incubator with 5% CO_2_. Valerate (#S844325) was purchased from Macklin. CGP35348 (#HY-103530) was purchased from MedChemExpress.

### Cell Stable Transfection

The coding sequences (CDS) of the human GABBR1 (NM_001470.4) and the mouse GABBR1 (NM_019439.4) were cloned into the lentiviral vectors pLenti-CMV-GFP-puro and pCDH-EF1-T2A-copGFP, respectively, generating the GABBR1 expression plasmid #PPL02876-4a (human) and #PPL50808-4a (mouse). Both plasmids were constructed and verified by Geneppl technology. Lentiviral particles were produced by co-transfecting HEK293T cells with the respective transfer vectors, the packaging plasmids psPAX2 (#PE081, Geneppl technology) and the envelope plasmid pMD2.G (#PE082, Geneppl technology) using Lipofectamine 3000 Transfection Reagent (#L3000001, Thermo Fisher Scientific). Viral supernatants were harvested at 48 h and 72 h post transfection. Lentiviral particles were concentrated by centrifugation (1500 xg, 30 min, 4 °C) using Amicon Ultra Centrifugal Filter, 100 kDa MWCO (#UFC9100, Merck). To establish stable cell lines, target cells at ~70% confluence were transduced with concentrated viral preparations in the presence of 8 μg/mL polybrene (Geneppl technology). After 12 hours, the viral supernatant was replaced with complete medium and subsequently selected with puromycin (2 μg/mL, # A1113803, Gibco) for 7 days. Surviving populations were validated for transgene expression by RT-qPCR and Western blot analysis.

### Animal Models

The 6-8 weeks old C57BL/6J mice and nude mice were reared in independently vented cages at the animal facility of the State Key Laboratory of Mechanism and Quality of Chinese Medicine, Macau University of Science and Technology, and Faculty of Health Sciences, University of Macau. All mice were acclimatized to the laboratory for one week before initiating the studies.

For valerate-treated animals, approximately 5×10^5^ Lewis lung cancer (LLC) cells were subcutaneously inoculated into the right flanks of C57BL/6J mice and nude mice, respectively. For the CD8^+^ T cell depletion mouse model, anti-CD8a antibody (200 μg/mouse; #BE0061, Bio X Cell) was administrated intraperitoneally every 3 days from day 0 and generated over the period. The control group was treated with PBS. Valerate (800 mg/kg/mouse) was intraperitoneally administrated injections twice daily from day 7 post-inoculation when tumor volumes were approximately 50 mm^3^. The tumor volumes and body weights were measured every 3 days.

For GABBR1-OE animal experiment, approximately 5×10^5^ LLC-LUC-GFP cells were orthotopically injected in the lung tissues as previously described 22. Body weight was determined every 7 days and, tumor volumes were monitored every 7 days by intraperitoneally injecting D-Luciferin potassium (150 mg/kg, 5 min before imaging; #ST196, Beyotime), followed by bioluminescence analysis using an *In Vivo* Imaging System (BLT Photon Technology).

All mice were humanely sacrificed after 21 days, and their tumors and tissues were dissected, weighed, and stored for subsequent experiments.

### Flow Cytometry

Flow cytometry analysis, immune single-cell suspensions were prepared from mouse blood and tumor tissues stained with following antibodies: PerCP anti-mouse CD45 (#103130, Biolegend), APC anti-mouse CD3 (#100236, Biolegend), FITC anti-mouse CD4 (#100406, Biolegend), PE/Cy7 anti-mouse CD8 (#100722, Biolegend), and PE/Dazzle 594 anti-mouse CD279 (PD-1; #135228, Biolegend). For intracellular staining, cells were stimulated with Cell Activation Cocktail (with Brefeldin A; #423303, Biolegend) for 4-6 h in the incubator at 37°C with 5% CO_2_. After being fixed and permeabilized, the cells were stained with APC/Cy7 anti-mouse interferon (IFN)-γ (#505826, Biolegend), PE/Dazzle 594 tumor necrosis factor (TNF)-α (#506346, Biolegend). For* in vitro* analyses, isolated CD8^+^ T cells were activated via CD3 (5 μg/mL)/CD28 (1 μg/mL) stimulation (#100340, #102116, Biolegend) along with 10 ng/mL of IL-2 (#575405, Biolegend). Flow cytometry analysis was performed using a CytoFLEX (Beckman Coulter) and data were analyzed using FlowJo software.

### Enzyme-linked Immunosorbent Detection (ELISA)

Mouse blood samples were centrifuged at 1000 g for 10 min to collect plasma. Cell culture medium was collected by centrifuging at 300 g for 5 min. The content of analytes IFN-γ, TNF-α, and cAMP was measured using ELISA kits according to the manufacturer's instructions. IFN-γ, TNF-α and cAMP were quantified using corresponding ELISA kits (#430804, #430904, Biolegend; #KGE012B, R&D System). GABA content was quantified using its specific ELISA kit (#OKEH02564, Aviva Systems Biology). The levels of analytes were quantified based on the standard curve generated for each assay. For each experiment, at least replicates were performed for each sample assay.

### Clonogenic Assay and Cell Proliferation Assay

For colony formation, A549 and H358 cells transfected with GABBR1-OE and wild type cells were seeded in 6-well plates and cultured for 9 days. Complete medium was replaced every 3 days for A549 cells and every 2 days for H358 cells. Colonies were fixed with 4% formaldehyde, stained with 0.5% crystal violet. The number of colonies was quantified using ImageJ software.

Cell proliferation was assessed using CCK-8 kit (#MA0218, MeilunBio). A549 and H358 cells were seeded in 96-well plates, and absorbance at 450 nm was measured daily for 6 days. Proliferation rates were normalized to Day 0 value.

Cell proliferation was further evaluated using BeyoClick™ EdU Cell Proliferation Kit with Alexa Fluor 594 (#C0078L, Beyotime). Following PBS washes, cells were incubated with EdU solution for 2 h (A549) or 6 h (H358). Cell nuclei were counterstained with DAPI solution. Fluorescent images were acquired using an inverted microscope (Leica) and EdU-positive cells were quantified.

### Human Samples Collection

The paraffin-embedded lung tissue specimens from 117 NSCLC patients were collected from 2014 to 2017 in Hubei Taihe Hospital. This study was approved by the Ethics Committee of Shiyan Taihe Hospital (2025KS148).

### Immunohistochemistry (IHC) Staining

Immunohistochemistry was performed using a commercial kit (#K8002; Dako) according to the manufacturer's protocol. Briefly, paraffin-embedded tissue sections were dewaxed in xylene, rehydrated through a graded ethanol series, and underwent antigen retrieval in substrate buffer (pH 6.0). Endogenous peroxidase activity was quenched by incubation in 3% hydrogen peroxide, followed by blocking non-specific binding with 5% BSA. Sections were subsequently incubated overnight at 4 °C with anti-GABA (#A2052, Sigma-Aldrich, 1:500) and anti-TNF-α (#ab215188, Abcam, 1:1000). After washing, the samples were incubated with the provided horseradish peroxidase (HRP) - conjugated secondary antibody and visualized using 3,3'-diaminobenzidine (DAB) chromogen as instructed. Cell nuclei were counterstained with Mayer's hematoxylin. Slides were dehydrated, cleared in xylene, and mounted.

### IHC Staining Evaluation

Stained slides were scanned and examined under a Leica optical microscope (Leica Microsystems). Clinical staining results were independently evaluated by two board-certificated pathologists blinded to sample identities. Staining intensity was scored as 0 (negative), 1 (slight brown), 2 (moderate brown), or 3 (dark brown). The staining area based on the positive cells was scored as 0 (0%), 1 (1%-25%), 2 (26%-50%), 3 (51%-75%), 4 (76%-100%). A final immunoreactivity score (IRS) was calculated by multiplying the intensity score by the area score. Protein expression levels were categorized based on IRS as follows: low (0-4), medium (5-8), or high (9-12). Mouse tissue staining results were analyzed using ImageJ software.

### Quantitative Real-Time PCR

Total RNA was isolated from cultured cells and tumor tissues using TRIzol reagent (#15596018CN, Invitrogen). RNA purity and concentration were determined by Nanodrop One (Thermo Fisher Scientific). cDNA was then synthesized using the iScript cDNA synthesis kit (#1708890, Bio-Rad). Quantitative real-time PCR (qPCR) was performed with PerfectStart Green qPCR SuperMix (#AQ602, TransGen Biotech), on CFX Opus 384 Real-Time PCR System (Bio-Rad), and the relative expression levels were analyzed using the 2-ΔΔCt method. The primer sequences used in this study are listed in Table S1.

### Western Blot

Protein was lysed using RIPA lysis buffer (#9806S, Cell Signaling Technology) with protease and phosphatase inhibitors (#46931, Roche, #78428, Thermo Fisher Scientific), followed by centrifuging and collecting the supernatant. The Bio-Rad Protein Assay Dye Reagent (#5000006, Bio-Rad) was used for protein concentration determination. Western blotting was performed with primary antibodies: anti-GABBR1 (#ab238130, Abcam, 1:800), anti-NRF2 (#12721, Cell Signaling Technology, 1:1000), anti-ATF-4 (#11815, Cell Signaling Technology, 1:1000), anti-NF-κB1 (p65) (#8242, Cell Signaling Technology, 1:1000), anti-NF-κB2 (p100/p52) (#4882, Cell Signaling Technology, 1:1000), anti-CXCL13 (#86564-1-RR, Proteintech, 1:1000), anti-p-AKT (#4060, Cell Signaling Technology, 1:1000), anti-AKT (#9272, Cell Signaling Technology, 1:1000), anti-p-GSK3β (#8566, Cell Signaling Technology, 1:1000), anti-GSK3β (#12456, Cell Signaling Technology, 1:1000), anti-GAPDH (#5174, Cell Signaling Technology, 1:1000), anti-ATP1A1 (#14418-1-AP, Proteintech, 1:15000) and anti-rabbit/mouse secondary antibody (#111-035-144 and #115-035-003, Jackson ImmunoResearch, 1:5000). The protein bands were visualized with Amersham ImageQuant 800 (Cytiva). Target protein expression levels were normalized to GAPDH.

### Molecular Docking Simulation

Molecular docking simulations were performed using the Schrödinger Suite (Schrödinger, LLC: New York, NY, 2024) to explore the binding mode of valeric acid and gamma-aminobutyric acid with the target enzyme. The crystal structure of Human GABBR1 (PDB ID: 4MQF 23) was retrieved from the Protein Data Bank (PDB) https://www.rcsb.org/. For docking analysis, Chain A of 4MQF was used as the receptor structure, and the binding site was defined based on the coordinates of the co-crystallized antagonist 2-hydroxysaclofen. The Protein Preparation Wizard module of the Schrödinger 2024 was employed to optimize the crystal structure, including the removal of all water molecules, the addition of missing hydrogen atoms, the addition of amino acid side chains, and generating the protonated state of GABBR1 at pH = 7.0 ± 2.0. Finally, the OPLS_2005^24^ force field was employed for constrained energy minimization of the GABBR1 structure until the root-mean-square deviation (RMSD) value was reduced to below 0.3 Å. Subsequently, the Receptor Grid Generation module of the Schrödinger 2024 was employed to generate a grid box for each complex centered on the natural ligand and defined as having a space size similar to that of the natural ligand.

The valeric acid, propanoic acid, butyric acid and gamma-aminobutyric acid were preprocessed using the LigPrep module of the Schrödinger package (LigPrep, Schrödinger, LLC, New York, NY, 2024). This preprocessing included the salt ion removal, charge neutralization and hydrogen atom optimization using the OPLS_2005 force field. The Epik module 24 was subsequently employed to calculate the possible ionization states of each compound at pH 7.0 ± 2.0, ensuring no tautomerism and preserving the original chirality of the compounds. Finally, molecular docking was performed using standard precision (SP) method with docking sites generated by the Glide module and processed ligands. The 2D receptor-ligand interactions were generated using Discovery Studio Visualizer 2020 (Accelrys), and visualization of the lowest energy conformations was performed with PyMOL 2.6 25.

### Public Dataset Collection

In this study, we acquired bulk RNA-seq datasets derived from lung cancer patients, including lung adenocarcinoma (LUAD) and squamous cell carcinoma (LUSC) via The Cancer Genome Atlas (TCGA) and corresponding healthy tissues from Genotype-Tissue Expression (GTEx) databases. Four single-cell RNA sequencing cohorts was obtained from the Gene Expression Omnibus (GEO) database, including GSE149655, GSE136246, GSE164829, GSE131097.

### Analysis of Immune Infiltration

To further explore the correlation between GABBR1 expression level and tumor immune infiltration, the abundance of tumor infiltration immune cells was calculated by three algorithms, including MCP-counter, EPIC, and Timer in the “IOBR” R package 26.

### Enrichment Analysis

GSEA was performed using the log2 (Foldchange) rank of all genes and calculated by DESeq2 method. The R package “clusterProfiler” 27 was used to perform Gene Ontology (GO), Kyoto Encyclopedia of Genes and Genomes (KEGG) and gene set enrichment analyses (GSEA). The Reactome gene set (m2.cp.reactome.v2024.1.Mm.entrez.gmt) and wiki gene set (m2.cp.wikipathways.v2024.1.Mm.entrez.gmt) were downloaded from the MSigDB database 28, and enrichment scores for each pathway were calculated for all samples.

### Single-cell RNA-Seq Data Processing

Four scRNA-seq datasets from above were processed separately using Seurat v5. Cells with more than 200 genes were pre-selected based on their expression profiles. The filtering criteria included a mitochondrial gene fraction of less than 10 %, a hemoglobin genes fraction of less than 3% and a ribosomal protein genes fraction of less than 50%. Post-selection, four datasets were integrated by “HarmonyIntegration” method and then re-normalized using Seurat's NormalizeData, and the topmost 2000 genes were selected as highly variable genes (HVG) and utilized to stabilize UMI count variance.

The principal component analysis (PCA) was performed using highly variable genes (HVGs), and the unified manifold approximation and projection (UMAP) was constructed using Louvain algorithm by selecting the first 30 principal components and clustering units. The major cell types were identified based on the marker genes from the published study: T cells were labeled with CD3D, CD3E, and CD3G; Natural killer cells (NKs) were labeled with CCL5, GNLY and NKG7; B cells were labeled with CD19, CD79A, and MS4A1; macrophages were labeled with APOE, CCL18, and TREM2; epithelial cells were labeled with KRT19, CLDN4 and EPCAM; monocytes were labeled with FCN1, VCAN and THBS1; fibroblasts were labeled with COL1A2, MFAP4, and LUM; endothelial cells were labeled with VWF, CLDN5, and PECAM1; plasma cells were labeled with IGKC, XBP1 and JCHAIN; dendritic cells were labeled with CLEC10A, CD1C, and CD86; mast cells were labeled with TPSAB1, TPSB2 and CPA3; and proliferating cells were labeled with TYMS, MKI67 and CCNA2.

To predict copy number alteration without tumor annotations, we used the CopyKAT 29 to define the aneuploid cell cluster.

### High-dimensional Weighted Gene Co-expression Network Analysis (hdWGCNA)

The hdWGCNA package is a tool designed for applying weighted gene co-expression network analysis (WGCNA) to investigate the genetic and biological characteristics of genes within high-dimensional gene expression datasets 30. We first preprocessed the gene expression data of malignant epithelial cells from lung adenocarcinoma using conventional methods, including quality control, normalization, and filtering of low-quality genes. Specifically, genes expressed in fewer than 2% of cells were removed, and the data were normalized using the “LogNormalize” method. Subsequently, we applied the hdWGCNA workflow to construct a weighted gene co-expression network, selecting a soft-thresholding power of 8 based on the scale-free topology criterion. The constructed gene co-expression network was further partitioned into distinct modules, each representing subnetworks composed of genes with highly similar expression patterns. From these, we identified modules with high module scores in the GABBR1-positive group and determined the intersection of the top 15 chemokines from each module to define candidate targets.

### Statistical Analysis

All data processing and statistical analyses were conducted using R software (versions 4.1.1). For comparisons between two groups, normally distributed data were analyzed using independent or unpaired Student's t-tests, while non-normally distributed data were evaluated using the Wilcoxon rank-sum test. For comparisons across multiple groups, one-way ANOVA, two-way ANOVA, or the Kruskal-Wallis's test was applied. Spearman's correlation and Pearson correlation analysis were used to calculate correlation coefficients. Overall survival was analyzed by log-rank. All statistical tests were two-sided, and statistical significance was set at P < 0.05, with additional thresholds reported as *p < 0.05, **p < 0.01, and ***p < 0.001.

## Results

### Valerate Exerts an *In Vivo* Anti-tumor Effect in Lung Cancer through Immune Response Activation

**Figure 1:**
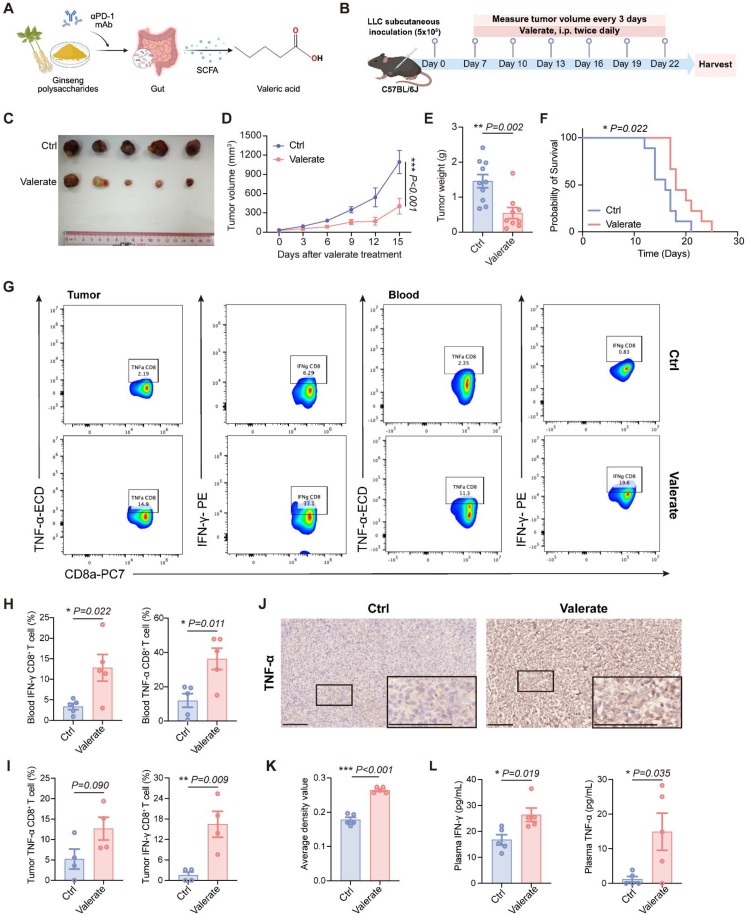
Valerate suppresses tumor growth in lung cancer by activating immune responses. A) Schematic diagram illustrating our previous work regarding valeric acid as a potential tumor suppressor and showing the structure of valeric acid. B) LLC-bearing C57BL/6J mice received intraperitoneal injections of valerate treatment (800 mg/kg) or PBS (control) twice daily from day 7 post-inoculation (n=9-10). All mice were sacrificed on day 22 for tumor collection and analysis. C) Representative images of the tumor on day 22 from valerate-treated groups and the control. D, E) Tumor volume growth curves and tumor weight between experimental groups. F) Kaplan-Meier curve indicating overall survival of tumor-bearing mice following tumor challenge between experimental groups (n=9 per groups). G) Representative results of flow cytometry analysis of cytokines (TNF-α, IFN-γ) in CD8^+^ T cells from tumors and blood, respectively. H, I) Quantitative analysis of flow cytometry data of blood and tumor between experimental groups. J) Representative IHC images of TNF-α expression in valerate-treated tumors and the controls (n=5 per group). Scale bar = 100 μm. K) Quantitative plots of average density of TNF-α in tumors tissues between experimental groups. L) Quantitative data of TNF-α and IFN-γ levels in the serum from mice between experimental groups. Data are presented as mean ± SEM. Statistical significance was determined by two-way ANOVA (D), log-rank (F) and unpaired t-test (E, H, I, K, L).

Previous work from our group revealed that microbial metabolite valeric acid levels were significantly elevated in lung cancer mouse models following combined treatment with ginseng polysaccharides and αPD-1 antibody 13. This finding suggests a potential role for valeric acid in modulating tumor progression and immune responses **(Figure 1A)**. To systematically evaluate valeric acid's anti-tumor efficacy, LLC-bearing C57BL/6J mice were subjected to daily intraperitoneal valerate (800 mg/kg) administration starting at day 7 post-inoculation. Tumor progression was monitored through longitudinal volume measurements and terminal weight analysis over a 15-day intervention period **(Figure 1B)**. Consistent with our hypothesis, valerate-treated mice exhibited significant tumor growth suppression compared to the control group **(Figure 1C-E)**. Correspondingly, the therapeutic effect markedly prolonged survival outcomes **(Figure 1F)**. In addition, there was no significant change in body weight between groups **(Figure S1)**. These results indicate that valerate exerts the anti-tumor effect in LLC-bearing mice.

To evaluate the immunomodulatory effects of valerate, we performed comprehensive peripheral and intratumoral immune profiles using flow cytometry. Peripheral blood analysis revealed that valerate treatment robustly activated CD8^+^ T cells, as evidenced by a significant increase in the production of cytotoxic effector molecules, IFN-γ and TNF-α, compared to untreated controls **(Figure 1G, H)**. In line with this, parallel examination of tumor-infiltrating lymphocytes (TILs) demonstrated valerate-induced enhancement of IFN-γ in CD8^+^ T cells, while TNF-α elevation showed a strong positive trend (*p=0.090*) **(Figure 1G, I)**. Additionally, we measured the cytokine levels in tumor tissues and plasma. Immunohistochemical staining revealed significant TNF-α accumulation within the tumor microenvironment of valerate-treated mice, consistent with enhanced cytotoxic T cell activity **(Figure 1J, K)**. Similarly, we observed notable increases in both IFN-γ and TNF-α secretion in plasma **(Figure 1L)**. Collectively, these findings establish that valerate exerts potent anti-tumor effects in lung cancer through inducing cytotoxic cytokines production and enhanced CD8^+^ T cell-mediated immune activation, demonstrating its therapeutic potential in lung cancer.

### Valerate Requires CD8^+^ T Cell Engagement to Mediate Anti-Tumor Responses

**Figure 2:**
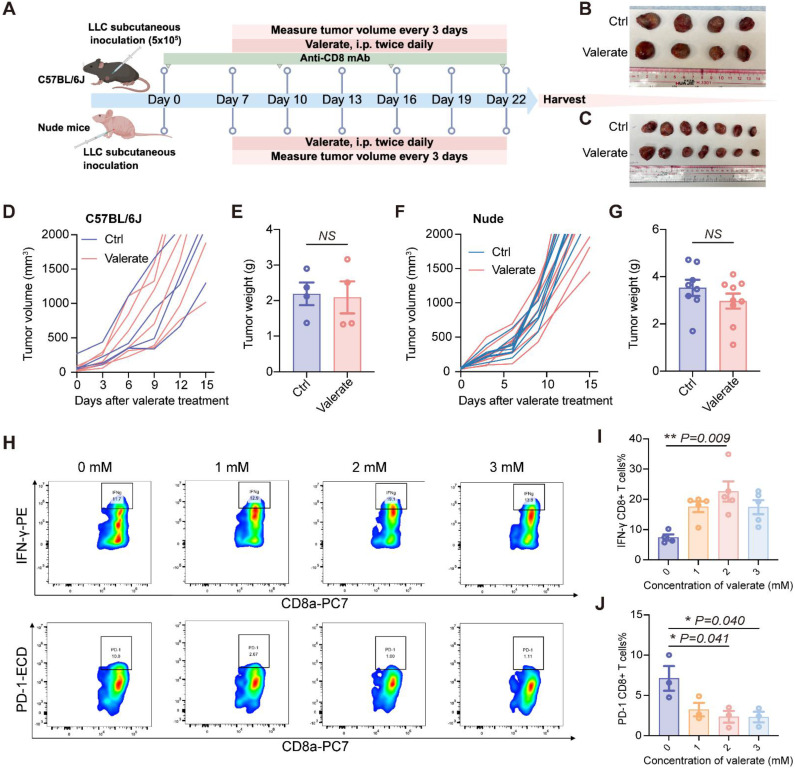
The anti-tumor activity of valerate is depending on CD8^+^ T cells. A) Schematic diagram of the *in vivo* experiment. C57BL/6J mice received subcutaneous injection of LLC cells followed by intraperitoneal treatment of valerate (800 mg/kg) on day 7. The CD8^+^ T cells were depleted by intraperitoneal injection of anti-CD8 antibody throughout the experiment (n=4 per group). Nude mice received subcutaneous injection of LLC cells followed by intraperitoneal treatment of valerate (800 mg/kg) on day 7 (n=8-9 per group). All mice were sacrificed on day 22 for tumor collection and analysis. B, C) Representative tumor images from C57BL/6J and nude mice, respectively. D, E) Tumor volume changes and tumor weight showed were from C57BL/J mice. F, G) Tumor volume change and tumor weight showed were from nude mice. H) Representative flow cytometry plots showing IFN-γ and PD-1 expression in CD8^+^ T cells treated with increasing concentrations of valerate for 24h *in vitro*. CD8^+^ T cells were freshly isolated from C57BL/6J mice spleen (n=5 for IFN-γ measurement, n=3 for PD-1 measurement). I, J) Quantitative analysis of flow cytometry data for IFN-γ (I) and PD-1 (J) expression. Data are presented as mean ± SEM. NS, not significant. Statistical significance was determined by two-tailed unpaired t-test (E, G) and one-way ANOVA (I, J).

To assess whether the anti-tumor effect is dependent on CD8^+^ T cells, we established CD8^+^ T cell depletion and CD8^+^ T cell deficient mouse models with LLC tumors **(Figure 2A)**. Consistent with our hypothesis that CD8^+^ T cells mediate the anti-tumor effect of valerate, depletion of CD8^+^ T cells completely abrogated valerate-induced tumor suppression. This was evidenced by the loss of inhibitory effect on tumor size **(Figure 2B, C)**, comparable tumor growth kinetics **(Figure 2D, F)** and tumor weights **(Figure 2E, G)** between valerate-treated and control groups. These data supported that CD8^+^ T cells as essential effectors responsible for the tumor-suppressive function of valerate.

To determine whether valerate directly activates CD8^+^ T cells, we purified CD8^+^ T cells from murine spleens and exposed them to escalating concentrations of valerate (0, 1, 2, 3 mM) **(Figure 2H)**. We found that 2 mM of valerate treatment triggered a substantial increase in secretion of IFN-γ by CD8^+^ T cells **(Figure 2I)**. Additionally, we examined the exhaustion markers and observed that valerate downregulated PD-1 in CD8^+^ T cells at higher concentrations (2-3 mM) **(Figure 2J)**, suggesting potential dose-dependent reduction of T cell exhaustion. Together, these findings indicate that valerate plays a potential role in maintaining CD8^+^ T cell activity and preventing exhaustion.

### Valerate Modulates GABBR1 Expression as a Binding Ligand

**Figure 3:**
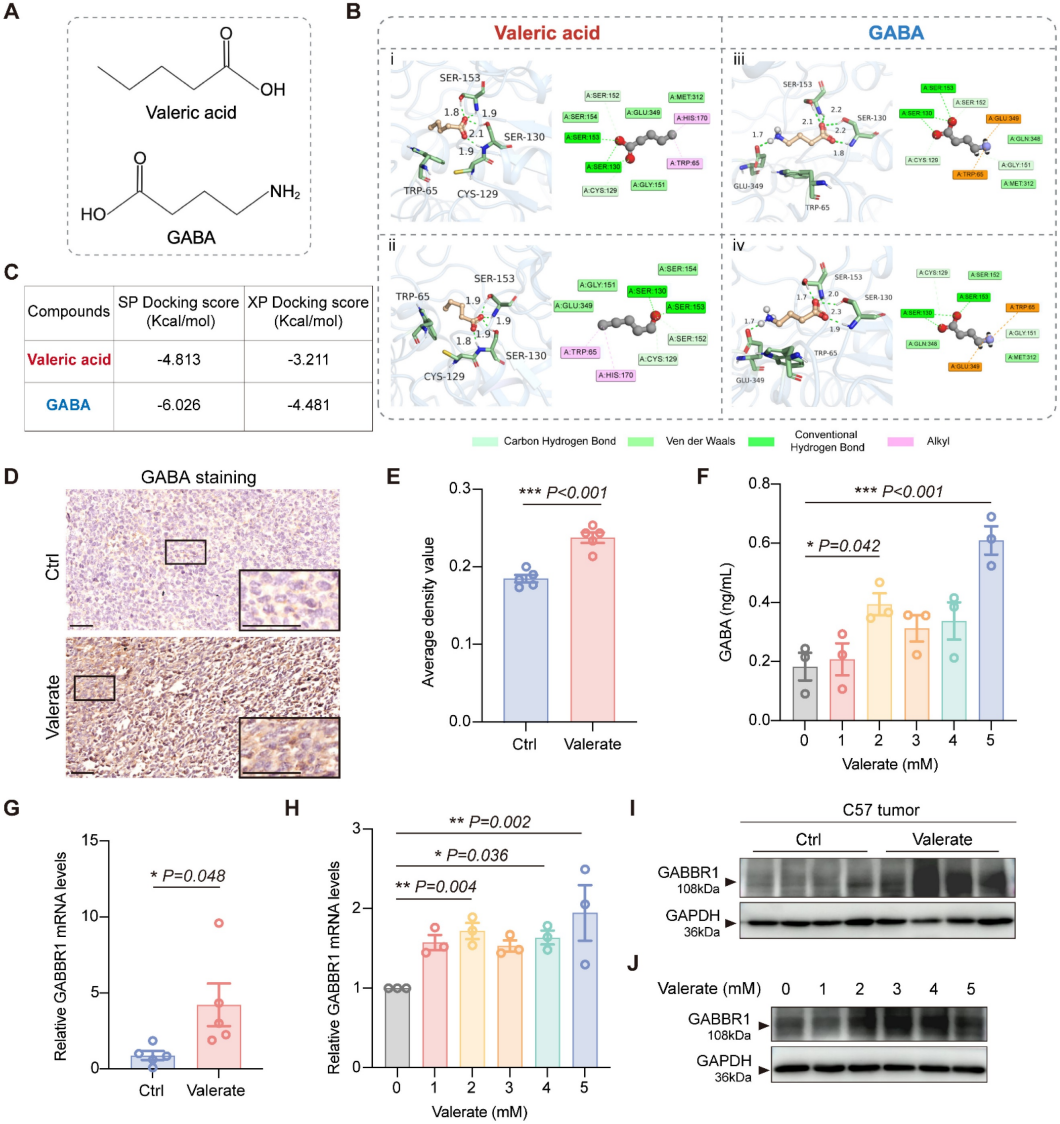
Valerate upregulates GABBR1 expression as a ligand. A) Chemical structures of valeric acid and GABA shown in this study. B) Three-dimensional (3D) and two-dimensional (2D) binding models of valeric acid or GABA with the GABBR1 protein generated through standard-precision (SP) molecular docking (i, iii) and extra-precision (XP) docking (ii, iv). C) SP docking and XP docking score of compounds valeric acid and GABA. D) Representative IHC images of GABA expression in valerate-treated tumors and the controls (n=5 per group). Scale bar = 50 μm. E) Quantitative analysis of GABA staining of tumor tissues between experimental groups. F) ELISA of GABA level in supernatants of LLC cells treated with elevated concentration of valerate at 24h after co-culture. G) RT-PCR analysis was conducted to measure the relative expression levels of GABBR1 in the tumors from valerate-treated mice and the controls (n=5 per group). H) RT-qPCR analysis of the relative expression levels of GABBR1 in the LLC cells treated with elevated concentration of valerate for 24h. I, J) Western blot analysis is performed to detect GABBR1 protein level in tumor tissues from valerate-treated mice and the controls (n=4 per group) (I) and in LLC cells treated with increasing concentration of valerate (0, 1, 2, 3, 4, 5 mM) (J). Data are presented as mean ± SEM. Statistical significance was determined by two-tailed unpaired t-test (E, G) and one-way ANOVA (F, H).

Based on the high structural similarity between valeric acid and GABA **(Figure 3A)** and given the reported involvement of GABA_B_ receptors in tumor development 31, 32. we hypothesized that valeric acid might also act as a ligand for GABA_B_ receptors. To verify this hypothesis, we performed molecular docking simulations to predict the binding interactions and affinities. Our result demonstrated that valeric acid interacts with key residues within the GABBR1 binding pocket, notably forming hydrogen bonds with Ser-130 and Ser-153 **(Figure 3Bi, Bii)**. The computed binding energies yield standard-precision (SP) score and extra-precision (XP) score of -4.813 kcal/mol and -3.211 kcal/mol, respectively **(Figure 3C)**. For reference, GABA - the native ligand of the receptor also formed hydrogen bonds with GABBR1 at Ser-130, Ser153, and Glu-349, with corresponding SP and XP scores of -6.026 kcal/mol and -4.481 kcal/mol **(Figure 3Biii, Biv, C)**. The comparable binding energies and overlapping interaction residues suggest that valeric acid can bind to the GABBR1 and may compete with GABA for receptor engagement. To assess the binding specificity among SCFAs, we also performed docking analysis for propionate and butyrate. The computed binding affinities (SP/XP scores) were lower than that of valerate (**Figure S2A, B**), indicating that valerate exhibits a superior binding potential to GABBR1 among these related metabolites.

To validate the computational prediction, we first examined GABA levels in mouse tumor specimens. Immunohistochemical (IHC) analysis showed a significant increase in overall GABA levels in valerate-treated tumors compared to controls **(Figure 3D, E)**, suggesting a potential impairment in the receptor-mediated clearance of GABA. To further investigate this accumulation, we treated LLC cells with valerate and observed a marked increase in the level of GABA in the culture medium **(Figure 3F)**. This result supports that valerate suppressed the internalization of GABA by competing for binding sites on the receptor. To rule out the possibility that elevated GABA levels resulted from altered metabolic flux, we examined the expression of key GABA metabolic enzymes (GAD65, ALDH5A1) and transporters (GAT1, GAT2, GAT4) in tumor tissues. No significant differences were observed between valerate-treated and control groups (**Figure S3A-C**), further supporting that GABA accumulation is primarily due to competitive inhibition of receptor binding.

To further confirm the effect of valerate on GABBR1, we first examined the expression levels of GABBR1 both *in vitro* and* in vivo*. We found that GABBR1 mRNA and protein expression was elevated in tumors from valerate-treated mice compared with controls **(Figure 3G, I)**. Consistent with this, treatment with valerate at gradient concentrations upregulated the GABBR1 expression in LLC cells **(Figure 3H, J)**. Further, we blocked GABBR1 using CGP35348, a specific competitive antagonist of the GABA_B_ receptor. The results showed that CGP35348 did not directly alter GABBR1 expression. Notably, the valerate-induced upregulation of GABBR1 was completely abolished upon co-treatment with CGP35348 **(Figure S3D)**. These results indicate that the enhancement of receptor expression by valerate is strictly dependent on binding to the active site of GABBR1, thereby ruling out potential off-target effects. Together, these findings demonstrate that valerate acts as a ligand for GABBR1 and upregulates its expression within the tumor microenvironment.

### Overexpression of GABBR1 Suppresses Cell Proliferation and Colony Formation in Lung Cancer

**Figure 4:**
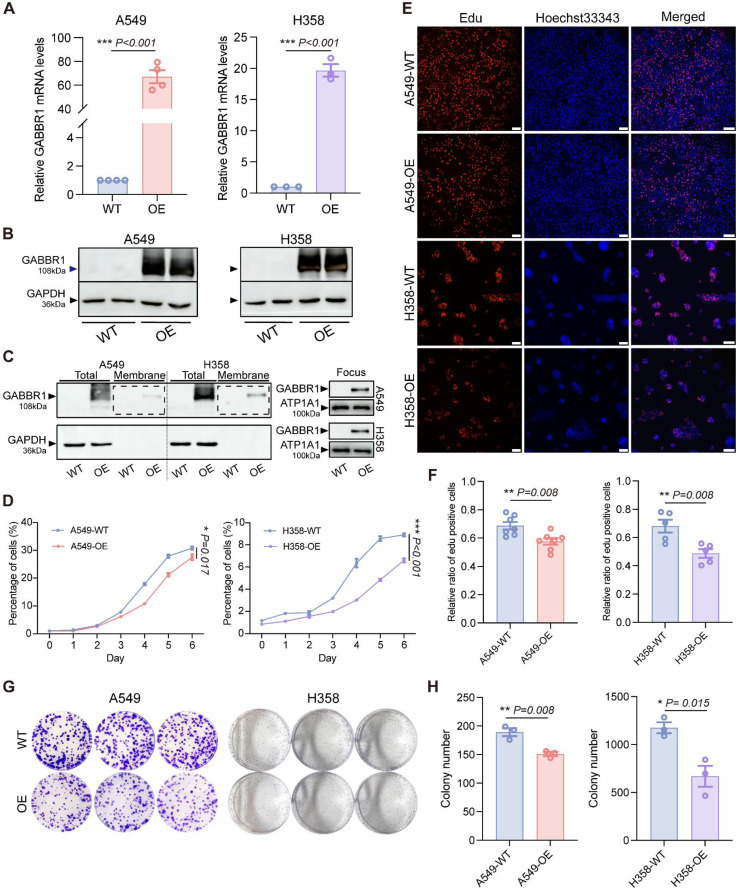
GABBR1 overexpression in cancer cells inhibited cell proliferation and colony formation ability. A, B) Overexpressing GABBR1 in A549 and H358 cells and determined by RT-qPCR (A) and western blot (B). C) Western blot analysis determined plasma membrane protein level of GABBR1 post-overexpression. D) CCK8 viability assay showing the cell proliferation for 6 days between the experimental groups. E) Representative images depicting EdU cell-cycle analysis after 3 h (A549) and 6 h (H358) when cells attached between experimental groups. Scale bar = 100 μm. F) Quantitative analysis of EdU-positive cells in A549 and H358 cells. G) Representative images of cell colony of control and GABBR1-overexpressing cells. H) Quantitative analysis of colony counts by image J software. Data are presented as mean ± SEM. Statistical significance was determined by two-tailed unpaired t-test (A, F, H) and two-way ANOVA (D).

To investigate the potential anti-tumor effect of GABBR1 mediated by valerate, we generated stable GABBR1-overepressing (GABBR1-OE) models in lung cancer cell lines A549 and H358 using a GABBR1-GFP expression plasmid. The overexpression efficiency was confirmed by RT-qPCR (mRNA level) and Western Blot (protein level) **(Figure 4A, B)**. Considering the functional requirement for plasma membrane localization of this heptahelical transmembrane receptor, we confirmed GABBR1 expression was also elevated in subcellular fractionation of overexpressing A549 and H358 cells by Western blotting **(Figure 4C)**. Additionally, to assess whether GABBR1 overexpression non-specifically affects its heterodimeric partner GABBR2, we examined GABBR2 expression levels and observed no significant changes (**Figure S4A, B**). Next, we evaluated the effect of GABBR1 overexpression on cell proliferation and colony formation. The suppression of cell proliferation mediated by overexpressing GABBR1 was assessed by CCK-8 assays over a period of 6 days. The result showed a significantly slower growth rate in GABBR1-OE cells compared to controls **(Figure 4D)**. Furthermore, the anti-proliferative effect was corroborated by EdU (5-ethynyl-2′-deoxyuridine) staining, which showed a marked reduction in DNA synthesis in GABBR1 overexpressing cells **(Figure 4E, F)**. To assess the long-term tumor suppressive capacity of GABBR1, we performed colony formation assays over 2 weeks in A549 and H358 cells. The colony counts indicated that GABBR1-OE cells exhibited a significant suppression clonogenic potential **(Figure 4G, H)**. Collectively, these findings indicate that GABBR1 plays a critical role in inhibiting cell proliferation and colony formation, thereby suppressing lung cancer progression.

### Overexpression of GABBR1 Suppresses Tumor Growth *In Vivo*

**Figure 5:**
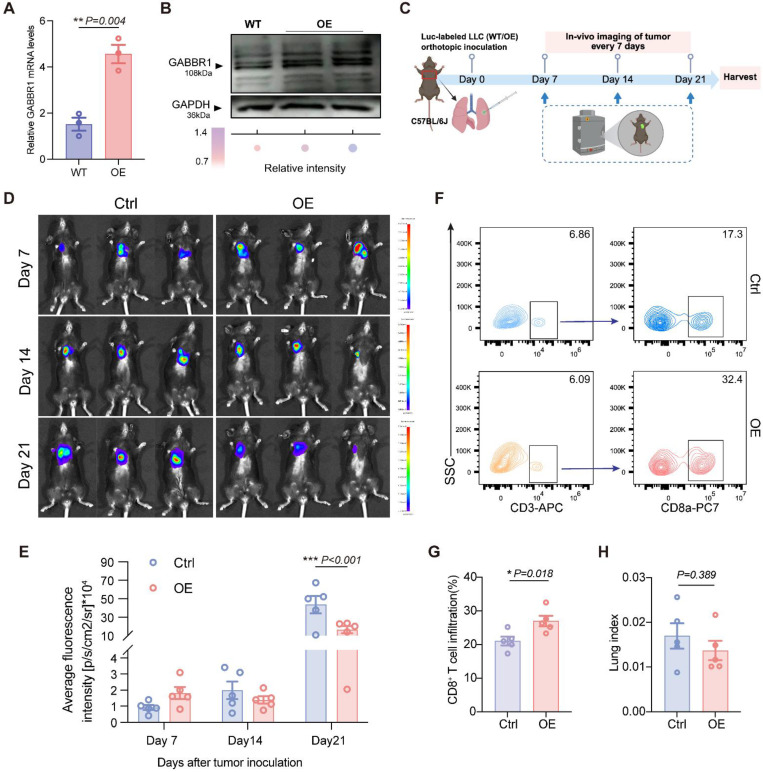
GABBR1 overexpression in tumor cells suppressed tumor growth with enhanced CD8^+^ T cells infiltration *in vivo*. Establishment and validation of stable GABBR1-overexpressing (GABBR1-OE) LLC-Luc cells. GABBR1 overexpression was confirmed by RT-qPCR (A) and Western blot (B), compared to wild-type (WT) controls. C) Schematic diagram of orthotopically injecting LLC-Luc (WT) or LLC-Luc (GABBR1-OE) cells in C57BL/6J mice. Tumor size was monitored longitudinally using *in vivo* bioluminescence imaging every 7 days. All mice were sacrificed on day 22 for tumor collection and analysis (n=5 per group). (D) Representative *in vivo* bioluminescence images of lung tumor at different time points post-injection. E) Quantitative analysis of bioluminescent intensity. F) Flow cytometry plots showing the tumor infiltrating CD3^+^ T cells and CD8^+^ T cells isolated from lung tumors from experimental groups. G) Quantitative analysis of infiltrated CD8^+^ T cells from flow cytometric result. H) The lung index (lung weight/body weight) calculated at endpoint. Data are presented as mean ± SEM. Statistical significance was determined by two-way ANOVA (E) and two-tailed unpaired t-test (A, G, H).

To evaluate the impact of GABBR1 on tumor progression *in vivo*, we first generated the GABBR1-OE LLC-Luc cell line and determined the transfection efficiency by RT-qPCR and Western blotting **(Figure 5A, B)**. GABBR2 expression levels remained unchanged, confirming the specificity of GABBR1 overexpression (**Figure S4C**). Subsequently, we established orthotopic xenograft tumor model with LLC-Luc WT and LLC-Luc-GABBR1-OE cells. Longitudinal monitoring of tumor progression was initiated from day 7 post-inoculation and evaluated at 7-day intervals using *in vivo* bioluminescence imaging **(Figure 5C)**. The results revealed that elevated GABBR1 levels markedly attenuated lung tumor progression, as evidenced by diminished average fluorescence intensity at the 21-day endpoint **(Figure 5D, E)**. In addition, no significant changes in body weight or other organ index (heart, thymus, kidney, liver, spleen) were detected between groups **(Figure S5A-F)**. To elucidate potential immunomodulatory mechanisms underlying tumor suppression, we conducted flow cytometry to characterize immune cell profiles. Our data showed that enhanced CD8^+^ T cell infiltration was detected in the tumor microenvironment in the GABBR1-OE group, consistent with the previous result **(Figure 5F, G)**. We also observed that the harvested lung tissues had smaller tumor areas (indicated by red curves) in the GABBR1-OE group compared to the control group **(Figure S5G)**. Although we observed a modest reduction in lung-to-body weight ratio, this limited discrepancy may be because the calculation relied mainly on tumor size rather than overall tumor burden **(Figure 5H)**. Taken together, these findings demonstrate that GABBR1-mediated tumor growth inhibition is mechanistically associated with enhanced cytotoxic T lymphocyte recruitment.

### GABBR1 Expression Correlates with Immune Infiltration and Chemokine Module Activation in LUAD

**Figure 6:**
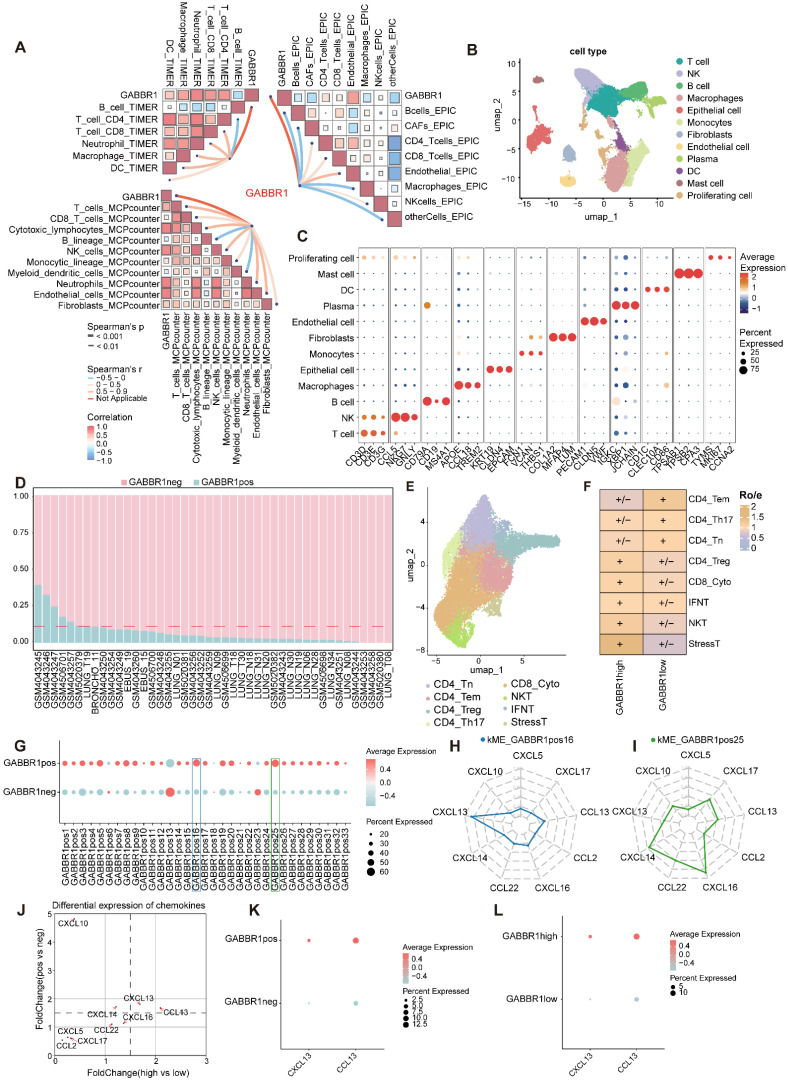
Comprehensive analysis of GABBR1 in bulk and single-cell RNA-seq datasets predict chemokines. A) The correlation between GABBR1 and the fraction of immune infiltration cells. B) A Uniform Manifold Approximation and Projection (UMAP) shows 112228 cells colored by major cell types. C) Dot plots show the average expression of known markers in indicated clusters. Dot size represents the proportion of cells with expression in each cluster and color indicates expression intensity. D) The ratio of GABBR1-pos/neg epithelial cells in patients. E) UMAP plot of subtypes of T cells. F) Heatmap showing the result of the Ro/e. G) Comparison of module eigengene scores between GABBR1pos and GABBR1neg groups. H-I) The core chemokines within GABBR1pos16 (H) and GABBR1pos25 (I) module were identified and prioritized based on their kME values. J) Differential expression of chemokines. Scatter plot of fold changes in chemokine expression between GABBR1-high vs low (x-axis) and GABBR1-pos vs neg (y-axis). K-L) CXCL13 and CCL13 expression levels were compared between GABBR1-pos vs neg (K) and GABBR1-high vs low (L).

To establish the overall relationship between GABBR1 expression and immune infiltration in LUAD, we first analyzed bulk RNA-seq data from the TCGA-LUAD cohort. Using three independent deconvolution algorithms (TIMER, MCP-counter, and EPIC), we consistently observed a positive correlation between GABBR1 expression and multiple immune cell populations, most notably CD8⁺ T cells **(Figure 6A)**. Consistently, GABBR1 expression also showed a positive correlation with CD8A expression at the bulk transcriptome level in the TCGA-LUAD cohort, further supporting an association between GABBR1 and cytotoxic T cell related signals **(Figure S6A)**. These findings suggest that GABBR1 may contribute to cytotoxic T cell recruitment in the tumor microenvironment. To further dissect this association at higher resolution, we integrated four single-cell RNA-seq datasets of lung adenocarcinoma, encompassing 112,228 cells across major immune and stromal populations **(Figure 6B, C, Figure S6B-E)**. Consistent with prior immunohistochemistry reports showing strong GABBR1 staining in tumor epithelium but negligible levels in immune or stromal compartments 33, our single-cell transcriptomic analysis confirmed that GABBR1 expression is predominantly enriched in malignant epithelial cells **(Figure S6F, G)**. Epithelial cells were classified as GABBR1-pos or neg based on detectable GABBR1 expression in single-cell RNA-seq data. For each patient, the proportion of GABBR1-pos epithelial cells was calculated, and samples in the top one-sixth were defined as GABBR1-high, with the remainder classified as GABBR1-low (**Figure 6D**). Notably, CD8⁺ cytotoxic T cells were significantly enriched in the GABBR1-high group as determined by the Ro/e algorithm, highlighting a potential link between epithelial GABBR1 expression and cytotoxic T cell infiltration **(Figure 6E, F, Figure S7A-C)**. To explore the transcriptional programs underlying this association, we applied the hdWGCNA framework to construct a weighted gene co-expression network within malignant epithelial cells **(Figure S8A, B)**. Modules were defined using a soft-thresholding power of 8 based on scale-free topology **(Figure S9)**. We then compared module eigengene scores between GABBR1-pos and GABBR1-neg groups and identified two modules, namely GABBR1pos16 and GABBR1pos25, that were significantly elevated in the GABBR1-pos subset **(Figure 6G)**. From these modules, we extracted the top 15 chemokines, and the intersection of these sets yielded nine candidate factors **(Figure 6H, I)**. Among them, CXCL13 and CCL13 were consistently enriched in both GABBR1-pos epithelial cells and GABBR1-high patients, highlighting their potential roles as mediators of GABBR1-associated immune infiltration **(Figure 6J-L, Table S2)**.

### Increased GABBR1 Level Induced by Valerate Stimulates CXCL13

**Figure 7:**
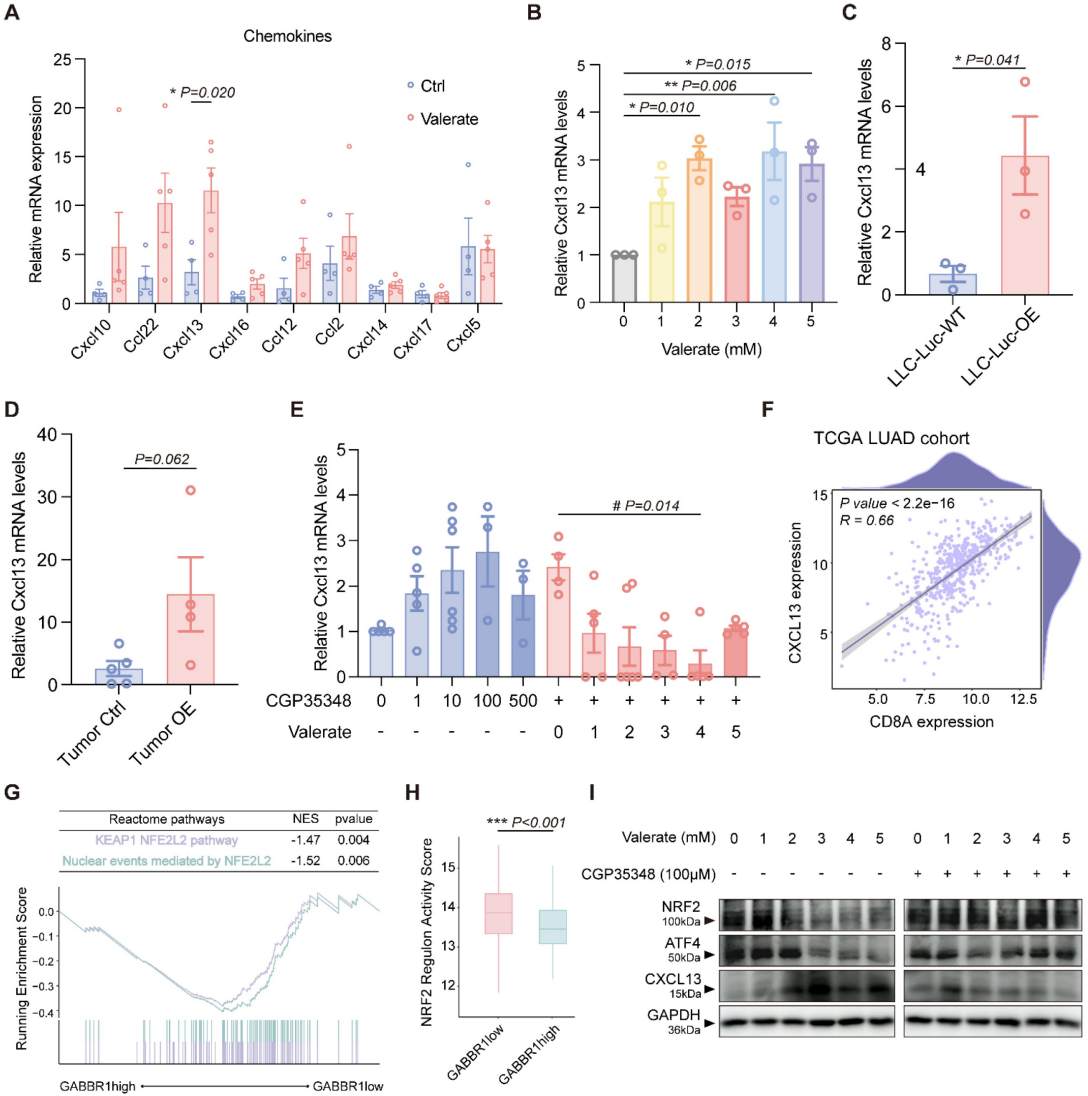
Valerate upregulates CXCL13 in the tumor microenvironment through GABBR1-ATF4-NRF2 axis. A) RT-qPCR analysis was conducted to measure the relative expression levels of the candidate chemokines in the tumors from valerate-treated mice and the controls (n=5 per group). B) RT-qPCR analysis of CXCL13 expression in LLC cells treated with elevated concentration of valerate (0, 1, 2, 3, 4, 5 mM) for 24 h. C, D) RT-qPCR analysis of CXCL13 expression in the wild type and GABBR1-OE LLC-Luc cells (C), and GABBR1-OE mice model (D) (n=5 per group). E) RT-qPCR analysis of CXCL13 in LLC cells treated with CGP35348 alone and co-treated with elevated concentration of valerate (0, 1, 2, 3, 4, 5 mM) and CGP35348 (100 μM) for 24h. F) Pearson correlation between CXCL13 and CD8A expression in tumors from patients in TCGA LUAD cohort. G) GSEA plot of significant Reactome pathways in comparison between the GABBR1high and GABBR1low groups in the TCGA LUAD cohort. H) Comparison of the regulon activity score of NRF2 between GABBR1 high and GABBR1 low groups in the TCGA LUAD cohort. I) Western blot analysis is performed to detect NRF2, ATF4 and CXCL13 protein levels in LLC cells treated with increasing concentration of valerate (0, 1, 2, 3, 4, 5 mM) with or without 100 μM of CGP35348. Data are presented as mean ± SEM. Statistical significance was determined by one way ANOVA (B, E), two-tailed unpaired t-test (A, C, D, H) and Pearson test (F).

To further validate the candidate chemokines (CXCL10, CCL22, CXCL13, CXCL16, CCL12, CCL2, CXCL14, CXCL17, CXCL5), we performed RT-qPCR to measure their expression levels in mouse tumor samples. Of note, because the mouse lacks a direct ortholog of human CCL13, we employed mouse CCL12 as a functional analog in the following measurement 34-37. Among these chemokines, CXCL13 and CCL12 were notably enriched in the GABBR1-pos cohort **(Figure 6J)**; however, only CXCL13 showed significant upregulation upon valerate treatment *in vivo* **(Figure 7A)**. To determine whether CXCL13 was directly regulated by valerate, we treated LLC cells with increasing concentrations of valerate and observed a dose-dependent induction of CXCL13 levels **(Figure 7B)**, confirming that exogenous valerate stimulates CXCL13 production in tumor cells. This result was aligned with our previous finding of valerate-induced GABBR1 levels **(Figure 3H)**, suggesting that GABBR1 may mediate valerate-induced CXCL13 expression.

To further investigate whether CXCL13 induction depends on GABBR1, we conducted RT-qPCR and observed a significant increase in CXCL13 expression in GABBR1-OE LLC cells **(Figure 7C)**, supporting the involvement of GABBR1 in this regulatory axis. In orthotopically tumor tissues, CXCL13 levels also showed an upward trend (*p=0.062*) **(Figure 7D)**. Notably, co-treatment with 100 μM CGP35348 *in vitro* reversed valerate-induced CXCL13 upregulation **(Figure 7E)**, demonstrating that GABBR1 was required for this effect. Besides, CGP35348 treatment alone did not affect GABBR1 expression, excluding as a potential confounding factor **(Figure S3D)**.

Subsequently, to explore the potential immunoregulatory mechanisms of CXCL13 induction, we first examined its relationship with CD8A expression. Analysis of TCGA-LUAD cohort revealed a strong positive correlation between CXCL13 and CD8A expression (R = 0.66, P < 2.2e-16) **(Figure 7F)**. Comparative GSEA of transcriptomes from GABBR1-high and GABBR1-low tumors revealed significant enrichment of NRF2-related Reactome pathways, including the KEAP1-NFE2L2 pathway and nuclear events mediated by NFE2L2 in the GABBR1-low group **(Figure 7G)**. Previous studies have identified NRF2 (NFE2L2) as a transcriptional repressor of CXCL13 38, suggesting that NRF2 activity may modulate CXCL13-mediated immune responses. Consistently, transcription factor activity profiling using the decoupleR package revealed significantly higher NRF2 regulatory activity in GABBR1-low tumors compared to GABBR1-high tumors **(Figure 7H)**.

To further confirm the effect of valerate on NRF2, we treated LLC cells with an elevated dose of valerate. As expected, the treatment resulted in a decrease in NRF2 levels. Additionally, ATF4 is a metabolic activator and co-factor of NRF2 that complexes with it to regulate downstream genes 39, 40. We also found that ATF4 was decreased when treated with valerate in a dose-dependent manner. ATF4 directly interacted with GABBR1 was confirmed by coimmunoprecipitation *in vivo* 41. This suppression was reversed by co-treatment with CGP35348** (Figure 7I)**, indicating that GABBR1 activity is essential for maintaining NRF2 expression. Consistently, valerate treatment also upregulated CXCL13 protein expression. as this effect was abrogated by CGP35348 co-treatment **(Figure 7I)**. Moreover, valerate treatment did not alter the activity of canonical GABBR1 downstream signaling pathways, including cAMP, AKT, and GSK3β, suggesting that the observed effects are independent of these classic cascades **(Figure S10A, B)**. Of note, CGP35348 treatment alone slightly elevated CXCL13 mRNA levels **(Figure 7E)**, but did not significantly elevate ATF4, NRF2 or CXCL13 protein levels, indicating that GABBR1 activation is required for full pathway engagement **(Figure S10C)**. Furthermore, *in vivo* valerate administration markedly upregulated CXCL13 protein expression in mouse tumor issues compared to controls, providing additional evidence that valerate promotes CXCL13 production via the GABBR1/ATF4/NRF2 axis **(Figure S10D)**. Together, these findings indicate that GABBR1 suppresses NRF2 activity, thereby depressing CXCL13 transcription and enhancing CD8⁺ T cell infiltration.

### GABA and GABBR1 Levels Predict Mortality in Lung Cancer Patient

**Figure 8:**
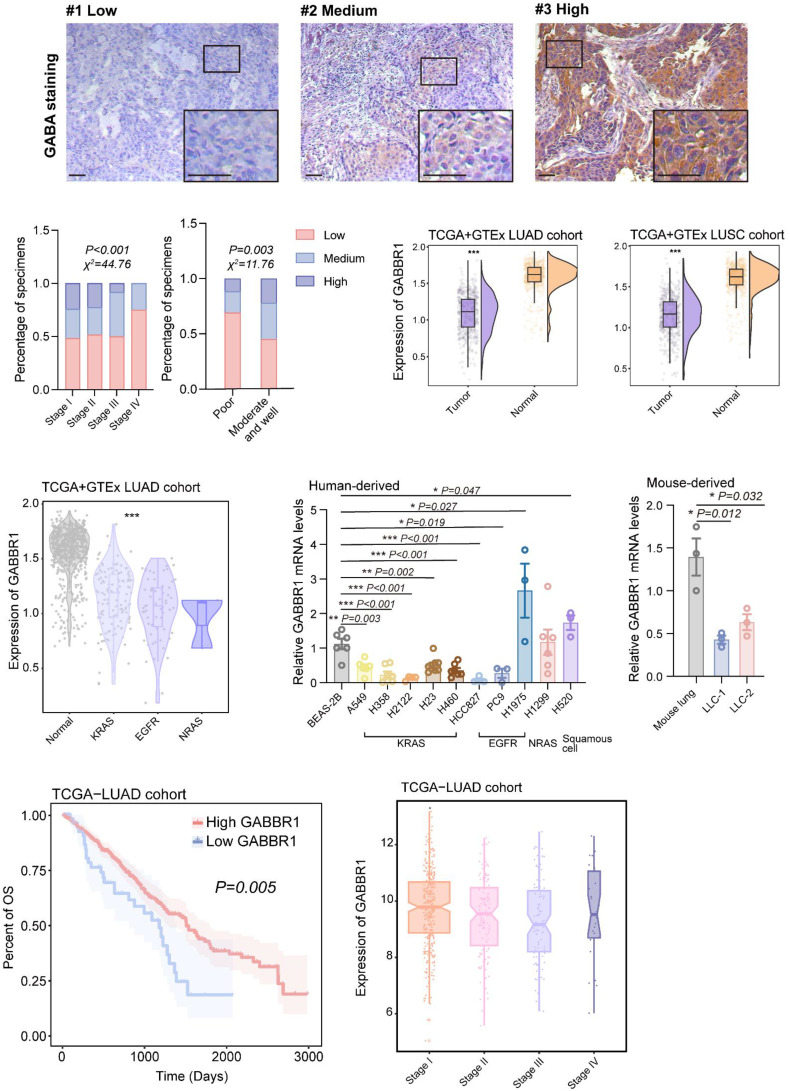
Clinical significance of GABBR1 as a promising therapeutic target to enhance anti-tumor immunity. A) Representative IHC images of GABA in clinical specimens from patients with lung cancer clarified as low, medium and high GABA (n=117). Scale bar =50 μm. B) Quantification of GABA staining of samples from patients with different clinical stages and cell differentiation of lung cancer. C) Analysis of GABBR1 expression in tumors and normal tissues in TCGA LUAD, TCGA LUSC and GTEx cohort. D) Analysis of GABBR1 expression in tumors harboring different mutations (KRAS, EGFR, NRAS) in TCGA LUAD and GTEx cohort. E, F) RT-qPCR analysis of GABBR1 in lung adenocarcinoma cell lines and lung squamous cell lines. Human lung cancer cell lines harboring different mutations (KRAS, EGFR, NRAS). Mouse lung cancer cell line compared to the mouse lung tissues (F). G) Kaplan-Meier survival analysis correlating GABBR1 expression in the LUAD cohort with survival probability. H) Quantification of GABBR1 staining of samples from patients in TCGA LUAD cohort Data are presented as mean ± SEM. Statistical significance was determined by χ2 tests (B), two-tailed unpaired t-test (C), long-rank (G), one way ANOVA (D, E, F, H).

To evaluate the clinical significance of GABA production across lung cancer progression stages, we performed IHC analysis on tumor specimens from a NSCLC cohort comprising 54 lung squamous cell carcinoma (LUSC) and 63 lung adenocarcinoma (LUAD) patients **(Figure 8A, Table S3)**. We found GABA was markedly enriched in early-stage (I-II) tumors compared to advanced-stage (III-IV) specimens, demonstrating progressive attenuation of GABA accumulation during disease progression **(Figure 8B)**. Notably, reduced GABA levels were strongly correlated with poor histological differentiation, whereas moderate-to-well-differentiated tumors exhibited higher GABA levels, indicating a potential association between GABA abundance and tumor cell differentiation status. Next, we assessed the clinical relevance of GABBR1. Integrated analysis of TCGA and GTEx datasets revealed significant downregulation of GABBR1 in NSCLC tumor tissues relative to adjacent normal tissues **(Figure 8C)**, corroborating its putative tumor-suppressive role as suggested by our earlier mechanistic studies. Furthermore, we observed that GABBR1 suppression was consistent across distinct mutation subtypes of LUAD **(Figure 8D)**. To this end, we verified the expression of GABBR1 in various lung cancer cell lines and found it was consistently downregulated in KRAS-mutant variants **(Figure 8E, F)**. Kaplan-Meier survival analysis demonstrated a striking inverse relationship between GABBR1 expression and mortality risk, as patients with high GABBR1 levels exhibited significantly prolonged overall survival compared to their low-expression counterparts **(Figure 8G)**. Longitudinal evaluation revealed progressive GABBR1 attenuation during tumor advancement, though a paradoxical increase was noted in stage IV specimens—a phenomenon potentially attributable to limited sample size in terminal-stage subgroups **(Figure 8H)**. Thus, these clinical findings establish GABA and its receptor GABBR1 as stage-dependent biomarkers with prognostic significance in lung cancer progression.

## Discussion and Conclusion

Increasing evidence suggests that SCFAs play a regulatory role in modulating responses to cancer therapy and are associated with the efficacy of diverse treatment regimens 42, 43. Here, we identify valerate as a promising anti-tumor molecule for NSCLC. Our results demonstrate that valerate significantly suppresses tumor progression by enhancing the infiltration and cytotoxic activity of CD8^+^ T cells, mediated through the upregulation of GABBR1. Mechanically, valerate acts as a ligand for GABBR1, leading to the inhibition of ATF4/NRF2 signaling axis and subsequent induction of CXCL13 expression. This chemokine facilitates robust CD8^+^ T cell recruitment into the tumor microenvironment, thereby exerting anti-tumor effects. These findings unveil a previously unrecognized role of valerate as a GABBR1 ligand and highlight its potential as an innovative strategy for lung cancer immunotherapy.

Valerate serves as a metabolic regulator that reprograms lymphocytes via mTOR signaling in CD4^+^ T cells and B cells, facilitating glucose oxidation, increasing acetyl-CoA levels and stimulating the release of immunosuppressive factor IL-10 44. In the context of cancer immunotherapy, our previous studies observed elevated valerate levels and increased cytotoxic factors in a combination treatment group receiving GPs and αPD-1 antibody in lung cancer, suggesting its therapeutic potential 13. This finding aligns with the work of Luu, M. *et al*., who demonstrated that valerate enhances CD8^+^ T cell function as a class I HDAC inhibitor, promoting the secretion of cytotoxic T lymphocytes (CTL)-associated factors such as TNF-α and IFN-γ, thereby potentially improving CAR-T cell efficacy 11. Consistent with these reports, our study further indicates that valerate treatment activates CD8^+^ T cells, increasing TNF-α and IFN-γ production while reducing PD-1 expression in lung cancer.

Although valerate and GABA are structurally similar, most studies have emphasized valerate's role as an HDAC inhibitor rather than its involvement in GABAergic signaling. However, recent reports using the drink-in-the-dark (DID) mouse model have shown that sodium valerate supplementation increases GABA levels both peripherally and in the brain 45. Additionally, valproic acid (VPA), a structural analog of valeric acid and an FDA-approved medication for absence seizures, enhances GABAergic signaling by inhibiting its degradation and stimulating its synthesis 46, 47. These observations provide evidence that valerate may also play a potential role in GABA regulation. Our findings verified that valerate promotes CD8^+^ T cell infiltration into microenvironment primarily acting as a ligand for GABBR1 rather than an HDAC inhibitor, unveiling a novel mechanism of valerate in modulating anti-tumor immunity.

Our immunohistochemical analysis revealed that higher levels of the endogenous ligand GABA are associated with well-differentiated tumors and lower malignancy, positioning GABA as a favorable biomarker and suggesting its role as an intrinsic regulator of tumor suppression. Notably, our functional compound valerate, while competing with GABA for receptor binding, does not counteract GABA's role but rather pharmacologically mimics and amplifies its protective signaling output. Thus, the therapeutic effect of valerate is aligned with the positive prognostic indication of high GABA levels observed in our clinical cohort. The decline of GABA in advanced stages may reflect a loss of this protective pathway, which can be therapeutically restored by valerate. Therefore, a combination of valerate treatment with chemotherapy/immunotherapy will be concluded in further investigation.

GABA_B_ receptor has been increasingly implicated in cancer pathogenesis. Previous studies in colorectal cancer demonstrate that the GABBR1 receptor activation impedes tumor progression by suppressing epithelial-mesenchymal transition (EMT) and inhibiting the Hippo/YAP signaling pathways 17. Consistent with this, GABA_B_ receptor activation has shown an inhibitory effect on cell proliferation and promoted cell apoptosis across several cancer types 19-21. In contrast, GABAergic signaling through the GABA_B_ receptor in turn promotes tumor growth by inhibiting CD8^+^ T cell activity 48. Thus, such controversies concerning the role of the GABA_B_ receptor have persisted. The concept of biased signaling provides a compelling framework for our findings. It is originally established and extensively characterized in G protein-coupled receptor (GPCR) studies, where different ligands binding to the same GPCR stabilize distinct conformation states, leading to the preferential activation of downstream signaling pathways 49, 50. The GABA_B_ receptor, a class C GPCR, recognizes ligands through the Venus flytrap (VFT) domain of the GABBR1 subunit 51. Our data revealed that valerate acts as a biased ligand that diverts GABA_B_ signaling toward a novel, anti-tumorigenic program. Once valerate binds to GABBR1, it triggers a distinct signaling pathway, specifically, inhibition of the ATF4/NRF2 axis and induction of CXCL13. Importantly, valerate administration did not cause significant adverse effects supporting its potential therapeutic safety 52 **(Figure 1F, S1)**. Further investigation should focus on solving the activate conformational state of the GABA_B_ receptor bound to valerate to elucidate the structural basis of its biased signaling properties.

Prior studies showed that the ATF4 directly interacts with GABA_B_ receptor, and the binding of ATF4 and GABBR2 to the GABBR1 subunit was mutually exclusive 53, 54. While GABBR1 is ubiquitously expressed in human tissues, GABBR2 expression is predominantly restricted to the central nervous system, suggesting that GABBR1 may exert functions independent of its heterodimerization partner GABBR2 55. In line with this, our results indicate that overexpression of GABBR1 was not accompanied by a significant increase in expression of GABBR2, implying that GABBR1 alone can form a functional unit (**Figure S4A-C**). This finding is also supported by several reports demonstrating that GABBR1 can traffic to the plasma membrane and form functionally active receptors in the absence of GABBR2 56, 57.

The interaction between ATF4 and GABBR1 promotes the nuclear translocation of ATF4 41. However, under conditions of sustained tumoral stress and prolonged receptor activation, we propose that nuclear accumulation of ATF4 may render it more susceptible to turnover mechanisms such as proteasomal degradation, which is in contrast to its canonical role initiating integrated stress response (ISR) 58, 59. This loss of ATF4 cripples the activation of NRF2 as ATF4 is recognized to stimulate NRF2 levels and transcriptional activity through its transcriptional factor CHAC1, which mediates glutathione degradation and subsequent NRF2 stabilization 39, 60. While NRF2 is in turn positively regulating ATF4 activity by increasing cystine uptake via the glutamate-cystine antiporter xCT 39, 40. Additionally, the high NRF2 activity confers a poor prognosis in human NSCLC 61. Interestingly, NRF2 also acts as a transcriptional repressor of CXCL13 38. In ovarian cancer, CXCL13 has been shown to promote anti-tumor immunity by sustaining CXCR5^+^CD8^+^ T cell populations within tertiary lymphoid structures (TLSs) and facilitating the recruitment of these cells to repair neuronal regeneration 62, 63. In our study, we demonstrated that the ATF4/NRF2/CXCL13 axis enhances the infiltration of CD8^+^ T cells towards tumor sites in lung cancer, uncovering a novel immunoregulatory mechanism in this malignancy.

In summary, our study demonstrated that valerate acts as a ligand for GABBR1 on tumor cells and enhances anti-tumor immunity by recruiting CD8^+^ T cells via ATF4/NRF2/CXCL13 signaling axis. Mechanistically, valerate-mediated suppression of NRF2 relieves its transcriptional repression of the chemokine CXCL13, leading to increased CXCL13 expression and enhanced CD8^+^ T cells in the tumor microenvironment. These findings nominate valerate as a novel and promising therapeutic candidate for the treatment of lung cancer.

## Supplementary Material

Supplementary methods, figures and tables.

## Data Availability

The data that support the findings of this study are available from the corresponding author, upon reasonable request.
