# Supplementary material for: Activation of GABBR1 by New Ligand Valerate Transcriptionally Regulates ATF4-NRF2-CXCL13 Axis Mediating CD8+ T Cell Anti-Tumor Immunity
Source: Int J Biol Sci. 2026 Apr 16;22(9):4496–516. doi: 10.7150/ijbs.127859 (PMC13181851; doi:10.7150/ijbs.127859)
Supplement: Supplementary file 1 — Supplementary methods, figures and tables. [file ijbsv22p4496s1.pdf]

Materials and Methods

Cell Culture and Treatment Compounds

The human NSCLC cell lines NCI-A549 (#CRM-CCL-185, RRID: CVCL_0023), NCI-H358 (#CRL-5807, RRID: CVCL_1559), NCI-H2122 (#CRL-5985, RRID: CVCL_1531), NCI-H23 (#CRL-5800, RRID: CVCL_1547), HCC827 (#CRL-2868, RRID: CVCL_2063), NCI-H1975 (#CRL-5908, RRID: CVCL1511), NCI-H1299 (#CRL-5803, RRID: CVCL_0060), the human large cell lung cancer NCI-H460 (#HTB-177, RRID: CVCL_0459), the human bronchial epithelial cell line BEAS-2B (#CRL-3588, RRID: CVCL_0168), the human embryonic kidney cell line HEK293T (CRL-3519, RRID: CVCL_0063), and the mouse Lewis lung carcinoma (LLC1; #CRL-1642, RRID: CVCL_4358) were purchased from the American Type Culture Collection (ATCC). The human NSCLC cell line PC-9 (#CL-0668, RRID: CVCL_B260) and squamous cell lung cancer cell line NCI-H520 (#CL-0402, RRID: CVCL_1566) were purchased from Procell Life Science & Technology. The LLC cells with luciferase and GFP marker (LLC-Luc-GFP; #STCC00071P) was purchased from Servicebio. All cell lines were tested and confirmed to be free of mycoplasma contamination. Primary $CD8^+$T cells were isolated and purified from murine spleen by using $CD8a^+$ T Cell Isolation Kit (#130-104-075, Miltenyi Biotec). A549, H358, H2122, H23, HCC827, H1975, H1299, H460 and H520 cells were cultured in RPMI 1640 Medium (#11875119, Gibco) supplemented with 10% of Fetal Bovine Serum (FBS; #10270106, Gibco) and 1% penicillin-streptomycin (100 U/mL; #15140122, Gibco). BEAS-2B, HEK293T, LLC and LLC-Luc-GFP cells were cultured in Dulbecco's Modified Eagle Medium (DMEM; #11965092, Gibco) supplemented with 10% FBS and 1% penicillin-streptomycin. All cell lines were cultured at 37°C in a humidified incubator with 5% CO_2. Valerate (#S844325) was purchased from Macklin. CGP35348 (#HY-103530) was purchased from MedChemExpress.

Cell Stable Transfection

The coding sequences (CDS) of the human GABBR1 (NM_001470.4) and the mouse GABBR1 (NM_019439.4) were cloned into the lentiviral vectors pLenti-CMV-GFP-puro and pCDH-EF1-T2A-copGFP, respectively, generating the GABBR1 expression plasmid #PPL02876-4a (human) and #PPL50808-4a (mouse). Both plasmids were constructed and verified by Geneppl technology. Lentiviral particles were produced by co-transfecting HEK293T cells with the respective transfer vectors, the packaging plasmids psPAX2 (#PE081, Geneppl technology) and

the envelope plasmid pMD2.G (#PE082, Geneppl technology) using Lipofectamine 3000 Transfection Reagent (#L3000001, Thermo Fisher Scientific). Viral supernatants were harvested at 48 h and 72 h post transfection. Lentiviral particles were concentrated by centrifugation (1500 xg, 30 min, 4°C) using Amicon Ultra Centrifugal Filter, 100 kDa MWCO (#UFC9100, Merck). To establish stable cell lines, target cells at ~70% confluence were transduced with concentrated viral preparations in the presence of 8 μg/mL polybrene (Geneppl technology). After 12 hours, the viral supernatant was replaced with complete medium and subsequently selected with puromycin (2 μg/mL, # A1113803, Gibco) for 7 days. Surviving populations were validated for transgene expression by RT-qPCR and Western blot analysis.

Animal Models

The 6-8 weeks old C57BL/6J mice and nude mice were reared in independently vented cages at the animal facility of the State Key Laboratory of Mechanism and Quality of Chinese Medicine, Macau University of Science and Technology, and Faculty of Health Sciences, University of Macau. All mice were acclimatized to the laboratory for one week before initiating the studies.

For valerate-treated animals, approximately 5×10^5 Lewis lung cancer (LLC) cells were subcutaneously inoculated into the right flanks of C57BL/6J mice and nude mice, respectively. For the $CD8^+$ T cell depletion mouse model, anti-CD8a antibody (200 μg/mouse; #BE0061, Bio X Cell) was administrated intraperitoneally every 3 days from day 0 and generated over the period. The control group was treated with PBS. Valerate (800 mg/kg/mouse) was intraperitoneally administrated injections twice daily from day 7 post-inoculation when tumor volumes were approximately 50 mm^3. The tumor volumes and body weights were measured every 3 days.

For GABBR1-OE animal experiment, approximately 5×10^5 LLC-LUC-GFP cells were orthotopically injected in the lung tissues as previously described [1]. Body weight was determined every 7 days and, tumor volumes were monitored every 7 days by intraperitoneally injecting D-Luciferin potassium (150 mg/kg, 5 min before imaging; #ST196, Beyotime), followed by bioluminescence analysis using an In Vivo Imaging System (BLT Photon Technology).

All mice were humanely sacrificed after 21 days, and their tumors and tissues were dissected, weighed, and stored for subsequent experiments.

Flow Cytometry

Flow cytometry analysis, immune single-cell suspensions were prepared from mouse blood and tumor tissues stained with following antibodies: PerCP anti-mouse CD45 (#103130, Biolegend), APC anti-mouse CD3 (#100236, Biolegend), FITC anti-mouse CD4 (#100406, Biolegend), PE/Cy7 anti-mouse CD8 (#100722, Biolegend), and PE/Dazzle 594 anti-mouse CD279 (PD-1; #135228, Biolegend). For intracellular staining, cells were stimulated with Cell Activation Cocktail (with Brefeldin A; #423303, Biolegend) for 4-6 h in the incubator at 37°C with 5% CO_2. After being fixed and permeabilized, the cells were stained with APC/Cy7 anti-mouse interferon (IFN)-γ (#505826, Biolegend), PE/Dazzle 594 tumor necrosis factor (TNF)-α (#506346, Biolegend). For *in vitro* analyses, isolated $CD8^+$ T cells were activated via CD3 (5 μg/mL)/CD28 (1 μg/mL) stimulation (#100340, #102116, Biolegend) along with 10 ng/mL of IL-2 (#575405, Biolegend). Flow cytometry analysis was performed using a CytoFLEX (Beckman Coulter) and data were analyzed using FlowJo software.

Enzyme-linked Immunosorbent Detection (ELISA)

Mouse blood samples were centrifuged at 1000 g for 10 min to collect plasma. Cell culture medium was collected by centrifuging at 300 g for 5 min. The content of analytes IFN-γ, TNF-α, and cAMP was measured using ELISA kits according to the manufacturer's instructions. IFN-γ, TNF-α and cAMP were quantified using corresponding ELISA kits (#430804, #430904, Biolegend; #KGE012B, R&D System). GABA content was quantified using its specific ELISA kit (#OKEH02564, Aviva Systems Biology). The levels of analytes were quantified based on the standard curve generated for each assay. For each experiment, at least replicates were performed for each sample assay.

Clonogenic Assay and Cell Proliferation Assay

For colony formation, A549 and H358 cells transfected with GABBR1-OE and wild type cells were seeded in 6-well plates and cultured for 9 days. Complete medium was replaced every 3 days for A549 cells and every 2 days for H358 cells. Colonies were fixed with 4% formaldehyde, stained with 0.5% crystal violet. The number of colonies was quantified using ImageJ software.

Cell proliferation was assessed using CCK-8 kit (#MA0218, MeilunBio). A549 and H358 cells were seeded in 96-well plates, and absorbance at 450nm was measured daily for 6 days. Proliferation rates were normalized to Day 0 value.

Cell proliferation was further evaluated using BeyoClick™ EdU Cell Proliferation Kit with Alexa Fluor 594 (#C0078L, Beyotime). Following PBS washes, cells were incubated with EdU solution for 2 h (A549) or 6 h (H358). Cell nuclei were counterstained with DAPI solution. Fluorescent images were acquired using an inverted microscope (Leica) and EdU-positive cells were quantified.

Human Samples Collection

The paraffin-embedded lung tissue specimens from 117 NSCLC patients were collected from 2014 to 2017 in Hubei Taihe Hospital. This study was approved by the Ethics Committee of Shiyan Taihe Hospital (2025KS148).

Immunohistochemistry (IHC) Staining

Immunohistochemistry was performed using a commercial kit (#K8002; Dako) according to the manufacturer's protocol. Briefly, paraffin-embedded tissue sections were dewaxed in xylene, rehydrated through a graded ethanol series, and underwent antigen retrieval in substrate buffer (pH 6.0). Endogenous peroxidase activity was quenched by incubation in 3% hydrogen peroxide, followed by blocking non-specific binding with 5% BSA. Sections were subsequently incubated overnight at 4°C with anti-GABA (#A2052, Sigma-Aldrich, 1:500) and anti-TNF-α (#ab215188, Abcam, 1:1000). After washing, the samples were incubated with the provided horseradish peroxidase (HRP) – conjugated secondary antibody and visualized using 3,3'-diaminobenzidine (DAB) chromogen as instructed. Cell nuclei were counterstained with Mayer's hematoxylin. Slides were dehydrated, cleared in xylene, and mounted.

IHC Staining Evaluation

Stained slides were scanned and examined under a Leica optical microscope (Leica Microsystems). Clinical staining results were independently evaluated by two board-certificated pathologists blinded to sample identities. Staining intensity was scored as 0 (negative), 1 (slight brown), 2 (moderate brown), or 3 (dark brown). The staining area based on the positive cells was scored as 0 (0%), 1 (1%-25%), 2 (26%-50%), 3 (51%-75%), 4 (76%-100%). A final immunoreactivity score (IRS) was calculated by multiplying the intensity score by the area score. Protein expression levels were categorized based on IRS as follows: low (0-4), medium (5-8), or high (9-12). Mouse tissue staining results were analyzed using ImageJ software.

Quantitative Real-Time PCR

Total RNA was isolated from cultured cells and tumor tissues using TRIzol reagent (#15596018CN, Invitrogen). RNA purity and concentration were determined by Nanodrop One (Thermo Fisher Scientific). cDNA was then synthesized using the iScript cDNA synthesis kit (#1708890, Bio-Rad). Quantitative real-time PCR (qPCR) was performed with PerfectStart Green qPCR SuperMix (#AQ602, TransGen Biotech), on CFX Opus 384 Real-Time PCR System (Bio-Rad), and the relative expression levels were analyzed using the $2^{-\Delta\Delta Ct}$ method. The primer sequences used in this study are listed in Table S1

Western Blot

Protein was lysed using RIPA lysis buffer (#9806S, Cell Signaling Technology) with protease and phosphatase inhibitors (#46931, Roche, #78428, Thermo Fisher Scientific), followed by centrifuging and collecting the supernatant. The Bio-Rad Protein Assay Dye Reagent (#5000006, Bio-Rad) was used for protein concentration determination. Western blotting was performed with primary antibodies: anti-GABBR1 (#ab238130, Abcam, 1:800), anti-NRF2 (#12721, Cell Signaling Technology, 1:1000), anti-ATF-4 (#11815, Cell Signaling Technology, 1:1000), anti-NF-κB1 (p65) (#8242, Cell Signaling Technology, 1:1000), anti-NF-κB2 (p100/p52) (#4882, Cell Signaling Technology, 1:1000), anti-CXCL13 (#86564-1-RR, Proteintech, 1:1000), anti-p-AKT (#4060, Cell Signaling Technology, 1:1000), anti-AKT (#9272, Cell Signaling Technology, 1:1000), anti-p-GSK3β (#8566, Cell Signaling Technology, 1:1000), anti-GSK3β (#12456, Cell Signaling Technology, 1:1000), anti-GAPDH (#5174, Cell Signaling Technology, 1:1000), anti-ATP1A1 (#14418-1-AP, Proteintech, 1:15000) and anti-rabbit/mouse secondary antibody (#111-035-144 and #115-035-003, Jackson ImmunoResearch, 1:5000). The protein bands were visualized with Amersham ImageQuant 800 (Cytiva). Target protein expression levels were normalized to GAPDH.

Molecular Docking Simulation

Molecular docking simulations were performed using the Schrödinger Suite (Schrödinger, LLC: New York, NY, 2024) to explore the binding mode of valeric acid and gamma-aminobutyric acid with the target enzyme. The crystal structure of Human GABBR1 (PDB ID: 4MQF [2]) was retrieved from the Protein Data Bank (PDB) https://www.rcsb.org/. For docking analysis, Chain A of 4MQF was used as the receptor structure, and the binding site was defined based on the coordinates of the co-crystallized antagonist 2-hydroxysaclofen. The Protein

Preparation Wizard module of the Schrödinger 2024 was employed to optimize the crystal structure, including the removal of all water molecules, the addition of missing hydrogen atoms, the addition of amino acid side chains, and generating the protonated state of GABBR1 at pH = 7.0 ± 2.0. Finally, the OPLS_2005[24] force field was employed for constrained energy minimization of the GABBR1 structure until the root-mean-square deviation (RMSD) value was reduced to below 0.3 Å. Subsequently, the Receptor Grid Generation module of the Schrödinger 2024 was employed to generate a grid box for each complex centered on the natural ligand and defined as having a space size similar to that of the natural ligand.

The valeric acid, propanoic acid, butyric acid and gamma-aminobutyric acid were preprocessed using the LigPrep module of the Schrödinger package (LigPrep, Schrödinger, LLC, New York, NY, 2024). This preprocessing included the salt ion removal, charge neutralization and hydrogen atom optimization using the OPLS_2005 force field. The Epik module [3] was subsequently employed to calculate the possible ionization states of each compound at pH 7.0 ± 2.0, ensuring no tautomerism and preserving the original chirality of the compounds. Finally, molecular docking was performed using standard precision (SP) method with docking sites generated by the Glide module and processed ligands. The 2D receptor-ligand interactions were generated using Discovery Studio Visualizer 2020 (Accelrys), and visualization of the lowest energy conformations was performed with PyMOL 2.6 [4].

Public Dataset Collection

In this study, we acquired bulk RNA-seq datasets derived from lung cancer patients, including lung adenocarcinoma (LUAD) and squamous cell carcinoma (LUSC) via The Cancer Genome Atlas (TCGA) and corresponding healthy tissues from Genotype-Tissue Expression (GTEx) databases. Four single-cell RNA sequencing cohorts was obtained from the Gene Expression Omnibus (GEO) database, including GSE149655, GSE136246, GSE164829, GSE131097.

Analysis of Immune Infiltration

To further explore the correlation between GABBR1 expression level and tumor immune infiltration, the abundance of tumor infiltration immune cells was calculated by three algorithms, including MCP-counter, EPIC, and Timer in the "IOBR" R package [5].

Enrichment Analysis

GSEA was performed using the log2 (Foldchange) rank of all genes and calculated by DESeq2 method. The R package “clusterProfiler” [6] was used to perform Gene Ontology (GO), Kyoto Encyclopedia of Genes and Genomes (KEGG) and gene set enrichment analyses (GSEA). The Reactome gene set (m2.cp.reactome.v2024.1.Mm.entrez.gmt) and wiki gene set (m2.cp.wikipathways.v2024.1.Mm.entrez.gmt) were downloaded from the MSigDB database [7], and enrichment scores for each pathway were calculated for all samples.

Single-cell RNA-Seq Data Processing

Four scRNA-seq datasets from above were processed separately using Seurat v5. Cells with more than 200 genes were pre-selected based on their expression profiles. The filtering criteria included a mitochondrial gene fraction of less than 10 %, a hemoglobin genes fraction of less than 3% and a ribosomal protein genes fraction of less than 50%. Post-selection, four datasets were integrated by “HarmonyIntegration” method and then re-normalized using Seurat’s NormalizeData, and the topmost 2000 genes were selected as highly variable genes (HVG) and utilized to stabilize UMI count variance.

The principal component analysis (PCA) was performed using highly variable genes (HVGs), and the unified manifold approximation and projection (UMAP) was constructed using Louvain algorithm by selecting the first 30 principal components and clustering units. The major cell types were identified based on the marker genes from the published study: T cells were labeled with CD3D, CD3E, and CD3G; Natural killer cells (NKs) were labeled with CCL5, GNLY and NKG7; B cells were labeled with CD19, CD79A, and MS4A1; macrophages were labeled with APOE, CCL18, and TREM2; epithelial cells were labeled with KRT19, CLDN4 and EPCAM; monocytes were labeled with FCN1, VCAN and THBS1; fibroblasts were labeled with COL1A2, MFAP4, and LUM; endothelial cells were labeled with VWF, CLDN5, and PECAM1; plasma cells were labeled with IGKC, XBP1 and JCHAIN; dendritic cells were labeled with CLEC10A, CD1C, and CD86; mast cells were labeled with TPSAB1, TPSB2 and CPA3; and proliferating cells were labeled with TYMS, MKI67 and CCNA2.

To predict copy number alteration without tumor annotations, we used the CopyKAT [8] to define the aneuploid cell cluster.

High-dimensional Weighted Gene Co-expression Network Analysis (hdWGCNA)

The hdWGCNA package is a tool designed for applying weighted gene co-expression network analysis (WGCNA) to investigate the genetic and biological characteristics of genes within

high-dimensional gene expression datasets [9]. We first preprocessed the gene expression data of malignant epithelial cells from lung adenocarcinoma using conventional methods, including quality control, normalization, and filtering of low-quality genes. Specifically, genes expressed in fewer than 2% of cells were removed, and the data were normalized using the "LogNormalize" method. Subsequently, we applied the hdWGCNA workflow to construct a weighted gene co-expression network, selecting a soft-thresholding power of 8 based on the scale-free topology criterion. The constructed gene co-expression network was further partitioned into distinct modules, each representing subnetworks composed of genes with highly similar expression patterns. From these, we identified modules with high module scores in the GABBR1-positive group and determined the intersection of the top 15 chemokines from each module to define candidate targets.

Statistical Analysis

All data processing and statistical analyses were conducted using R software (versions 4.1.1). For comparisons between two groups, normally distributed data were analyzed using independent or unpaired Student's t-tests, while non-normally distributed data were evaluated using the Wilcoxon rank-sum test. For comparisons across multiple groups, one-way ANOVA, two-way ANOVA, or the Kruskal-Wallis's test was applied. Spearman's correlation and Pearson correlation analysis were used to calculate correlation coefficients. Overall survival was analyzed by log-rank. All statistical tests were two-sided, and statistical significance was set at $P < 0.05$, with additional thresholds reported as *$p < 0.05$, **$p < 0.01$, and ***$p < 0.001$.

Figures

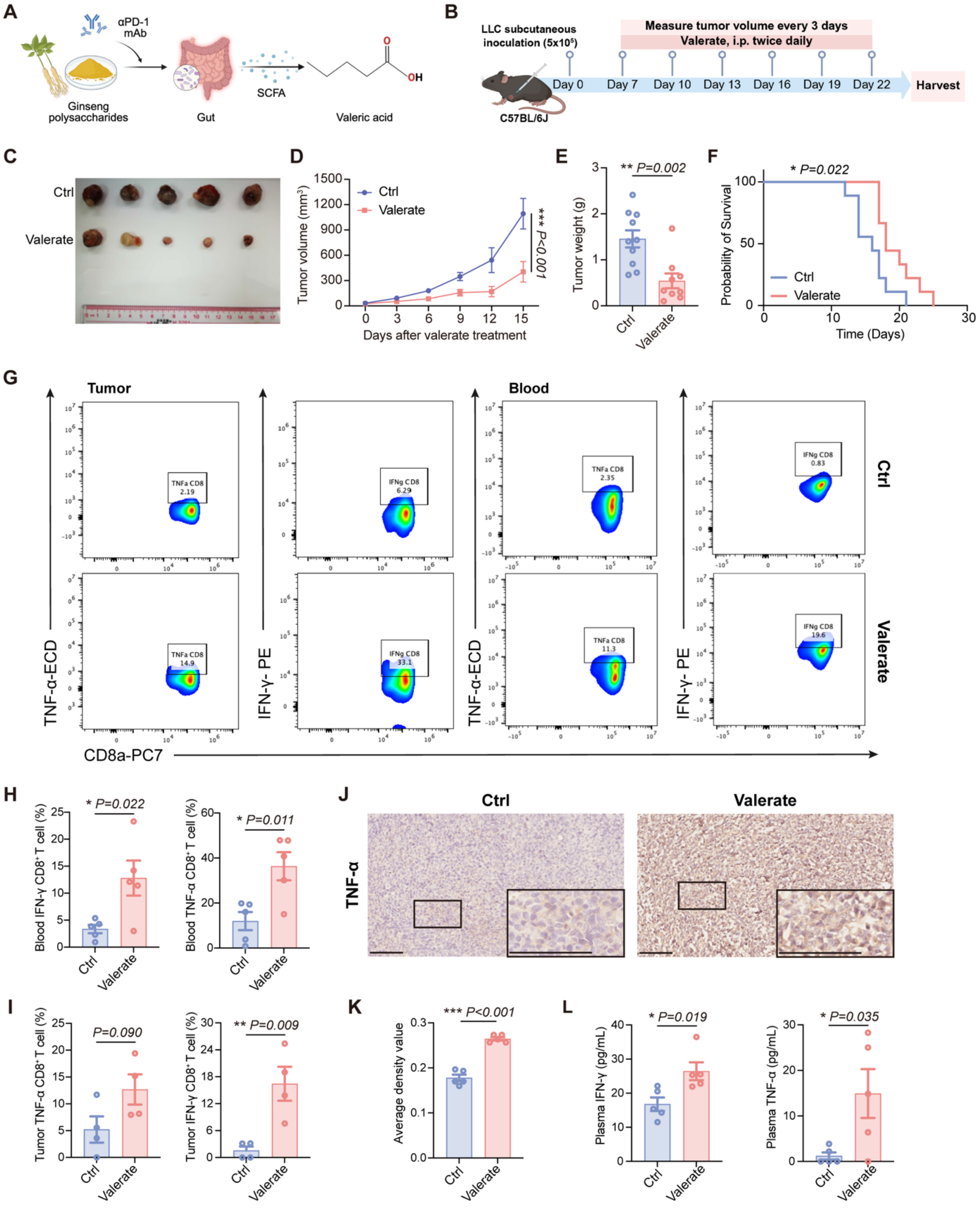

Figure 1 Valerate suppresses tumor growth in lung cancer by activating immune responses. A) Schematic diagram illustrating our previous work regarding valeric acid as a potential tumor suppressor and showing the structure of valeric acid. B) LLC-bearing C57BL/6J mice received intraperitoneal injections of valerate treatment (800 mg/kg) or PBS (control) twice daily from

day 7 post-inoculation (n=9-10). All mice were sacrificed on day 22 for tumor collection and analysis. C) Representative images of the tumor on day 22 from valerate-treated groups and the control. D, E) Tumor volume growth curves and tumor weight between experimental groups. F) Kaplan-Meier curve indicating overall survival of tumor-bearing mice following tumor challenge between experimental groups (n=9 per groups). G) Representative results of flow cytometry analysis of cytokines (TNF-α, IFN-γ) in $CD8^+$ T cells from tumors and blood, respectively. H, I) Quantitative analysis of flow cytometry data of blood and tumor between experimental groups. J) Representative IHC images of TNF-α expression in valerate-treated tumors and the controls (n=5 per group). Scale bar = 100 μm. K) Quantitative plots of average density of TNF-α in tumors tissues between experimental groups. L) Quantitative data of TNF-α and IFN-γ levels in the serum from mice between experimental groups. Data are presented as mean ± SEM. Statistical significance was determined by two-way ANOVA (D), log-rank (F) and unpaired t-test (E, H, I, K, L).

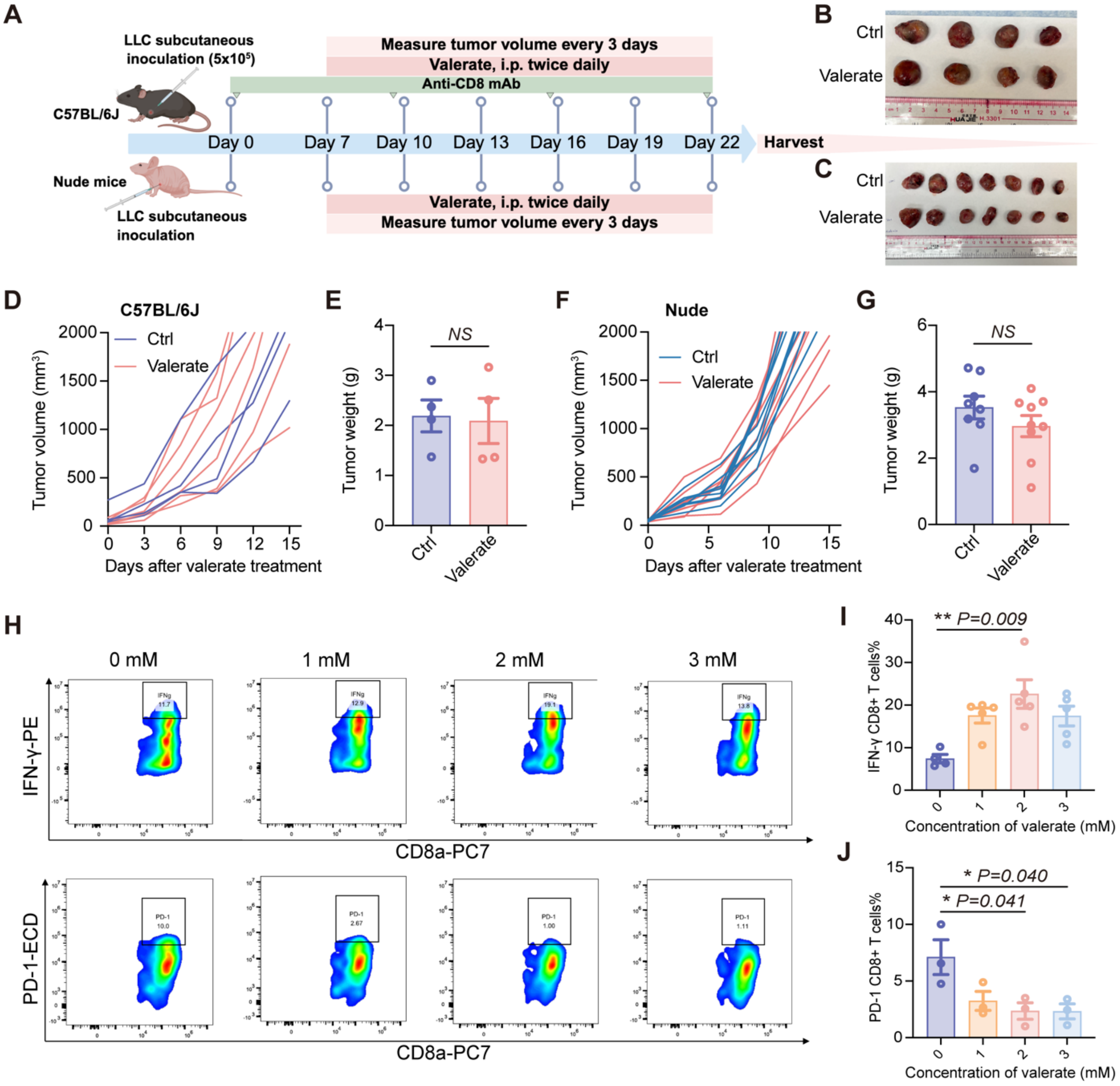

Figure 2 The anti-tumor activity of valerate is depending on CD8$^+$ T cells. A) Schematic diagram of the *in vivo* experiment. C57BL/6J mice received subcutaneous injection of LLC cells followed by intraperitoneal treatment of valerate (800 mg/kg) on day 7. The CD8$^+$ T cells were depleted by intraperitoneal injection of anti-CD8 antibody throughout the experiment (n=4 per group). Nude mice received subcutaneous injection of LLC cells followed by intraperitoneal treatment of valerate (800 mg/kg) on day 7 (n=8-9 per group). All mice were sacrificed on day 22 for tumor collection and analysis. B, C) Representative tumor images from C57BL/6J and nude mice, respectively. D, E) Tumor volume changes and tumor weight showed were from C57BL/J mice. F, G) Tumor volume change and tumor weight showed were from nude mice. H) Representative flow cytometry plots showing IFN-γ and PD-1 expression in CD8$^+$ T cells treated with increasing concentrations of valerate for 24h *in vitro*. CD8$^+$ T cells were freshly isolated from C57BL/6J mice spleen (n=5 for IFN-γ measurement, n=3 for PD-1 measurement).

I, J) Quantitative analysis of flow cytometry data for IFN-γ (I) and PD-1 (J) expression. Data are presented as mean ± SEM. NS, not significant. Statistical significance was determined by two-tailed unpaired t-test (E, G) and one-way ANOVA (I, J).

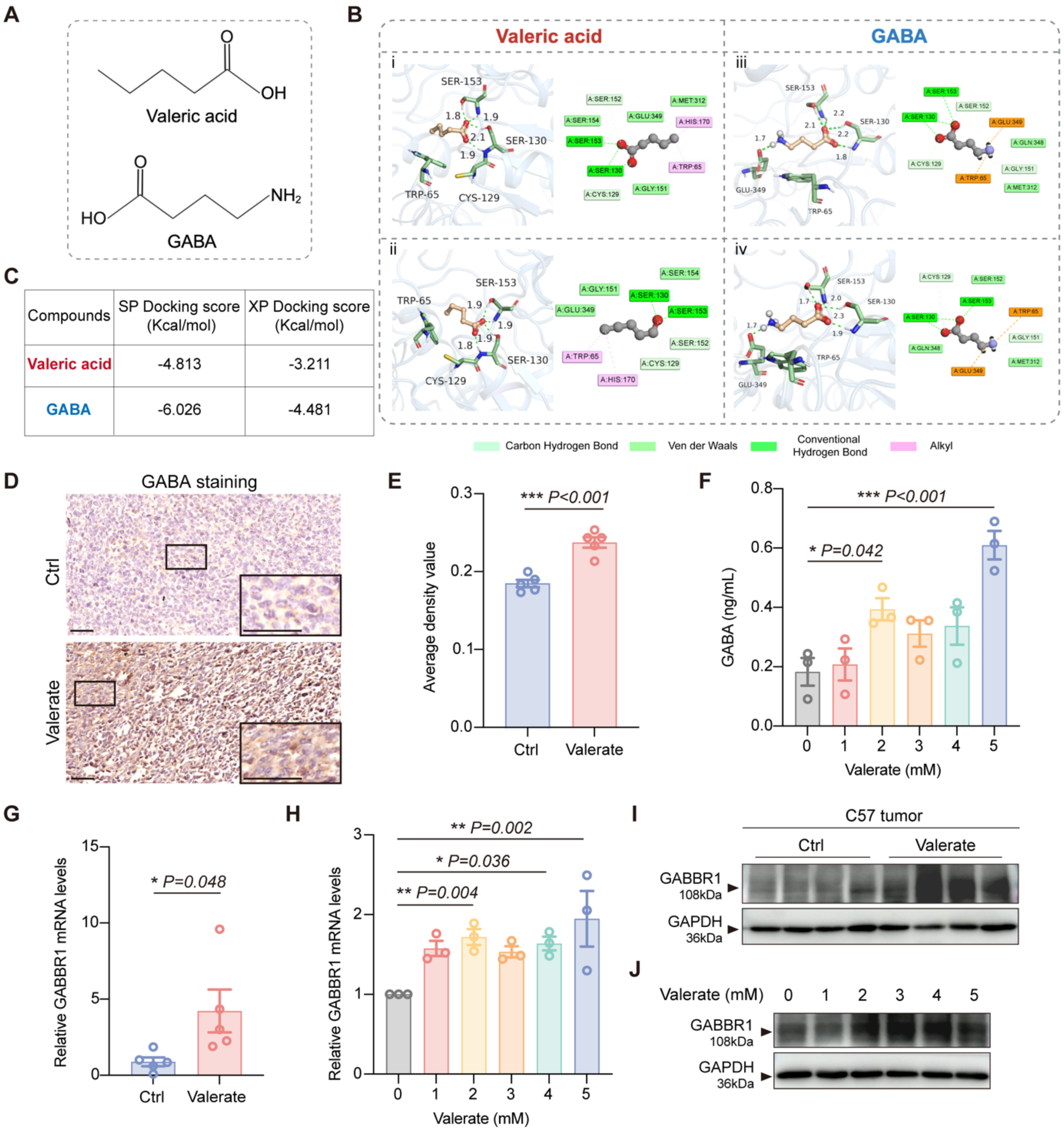

Figure 3 Valerate upregulates GABBR1 expression as a ligand. A) Chemical structures of valeric acid and GABA shown in this study. B) Three-dimensional (3D) and two-dimensional (2D) binding models of valeric acid or GABA with the GABBR1 protein generated through standard-precision (SP) molecular docking (i, iii) and extra-precision (XP) docking (ii, iv). C) SP docking and XP docking score of compounds valeric acid and GABA. D) Representative IHC images of GABA expression in valerate-treated tumors and the controls (n=5 per group). Scale bar = 50 μm. E) Quantitative analysis of GABA staining of tumor tissues between experimental groups. F) ELISA of GABA level in supernatants of LLC cells treated with elevated concentration of valerate at 24h after co-culture. G) RT-PCR analysis was conducted to measure the relative expression levels of GABBR1 in the tumors from valerate-treated mice

and the controls (n=5 per group). H) RT-qPCR analysis of the relative expression levels of GABBR1 in the LLC cells treated with elevated concentration of valerate for 24h. I, J) Western blot analysis is performed to detect GABBR1 protein level in tumor tissues from valerate-treated mice and the controls (n=4 per group) (I) and in LLC cells treated with increasing concentration of valerate (0, 1, 2, 3, 4, 5 mM) (J). Data are presented as mean ± SEM. Statistical significance was determined by two-tailed unpaired t-test (E, G) and one-way ANOVA (F, H).

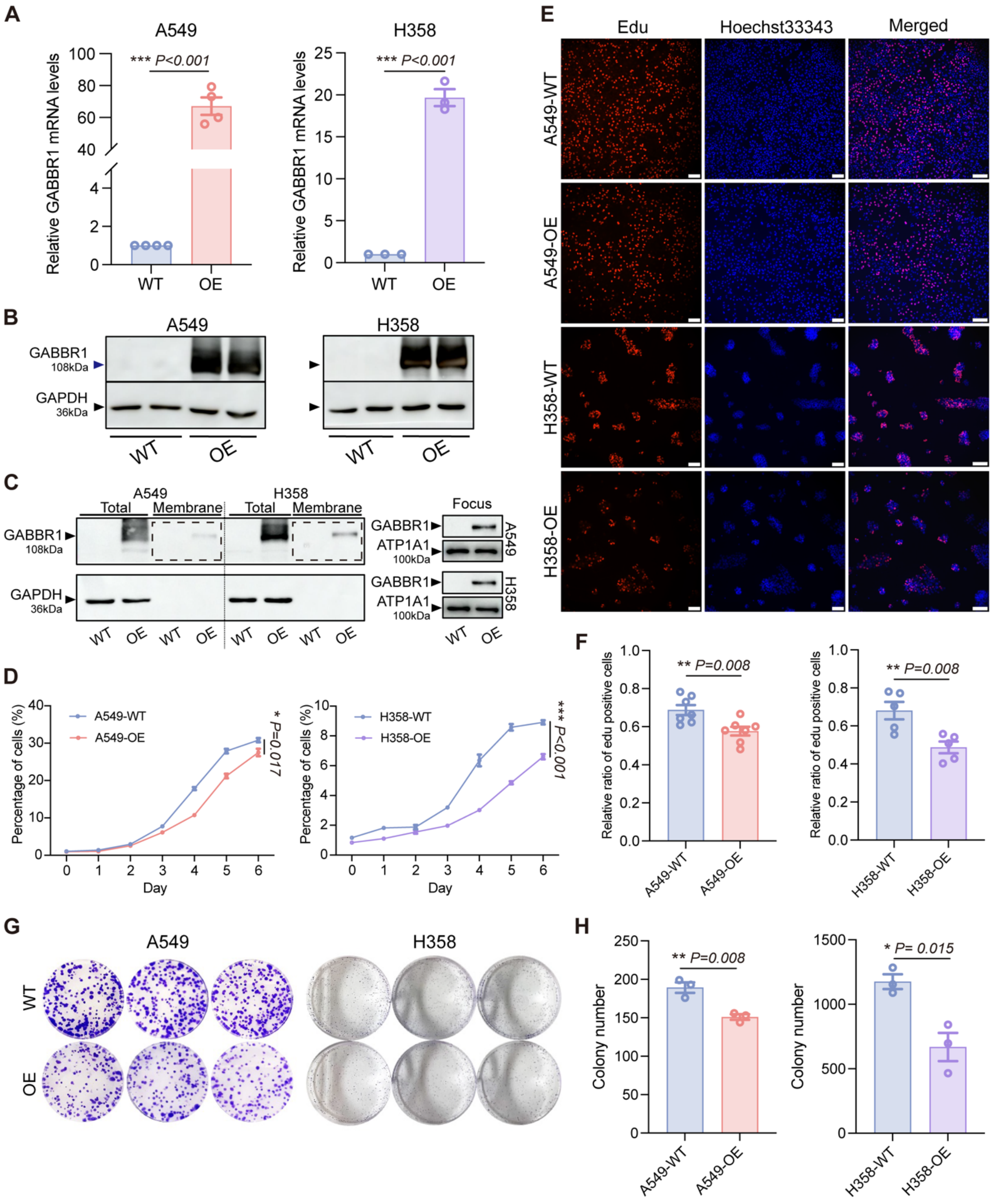

Figure 4 GABBR1 overexpression in cancer cells inhibited cell proliferation and colony formation ability. A, B) Overexpressing GABBR1 in A549 and H358 cells and determined by RT-qPCR (A) and western blot (B). C) Western blot analysis determined plasma membrane protein level of GABBR1 post-overexpression. D) CCK8 viability assay showing the cell proliferation for 6 days between the experimental groups. E) Representative images depicting EdU cell-cycle analysis after 3 h (A549) and 6 h (H358) when cells attached between

experimental groups. Scale bar = 100 μm. F) Quantitative analysis of EdU-positive cells in A549 and H358 cells. G) Representative images of cell colony of control and GABBR1-overexpressing cells. H) Quantitative analysis of colony counts by image J software. Data are presented as mean ± SEM. Statistical significance was determined by two-tailed unpaired t-test (A, F, H) and two-way ANOVA (D).

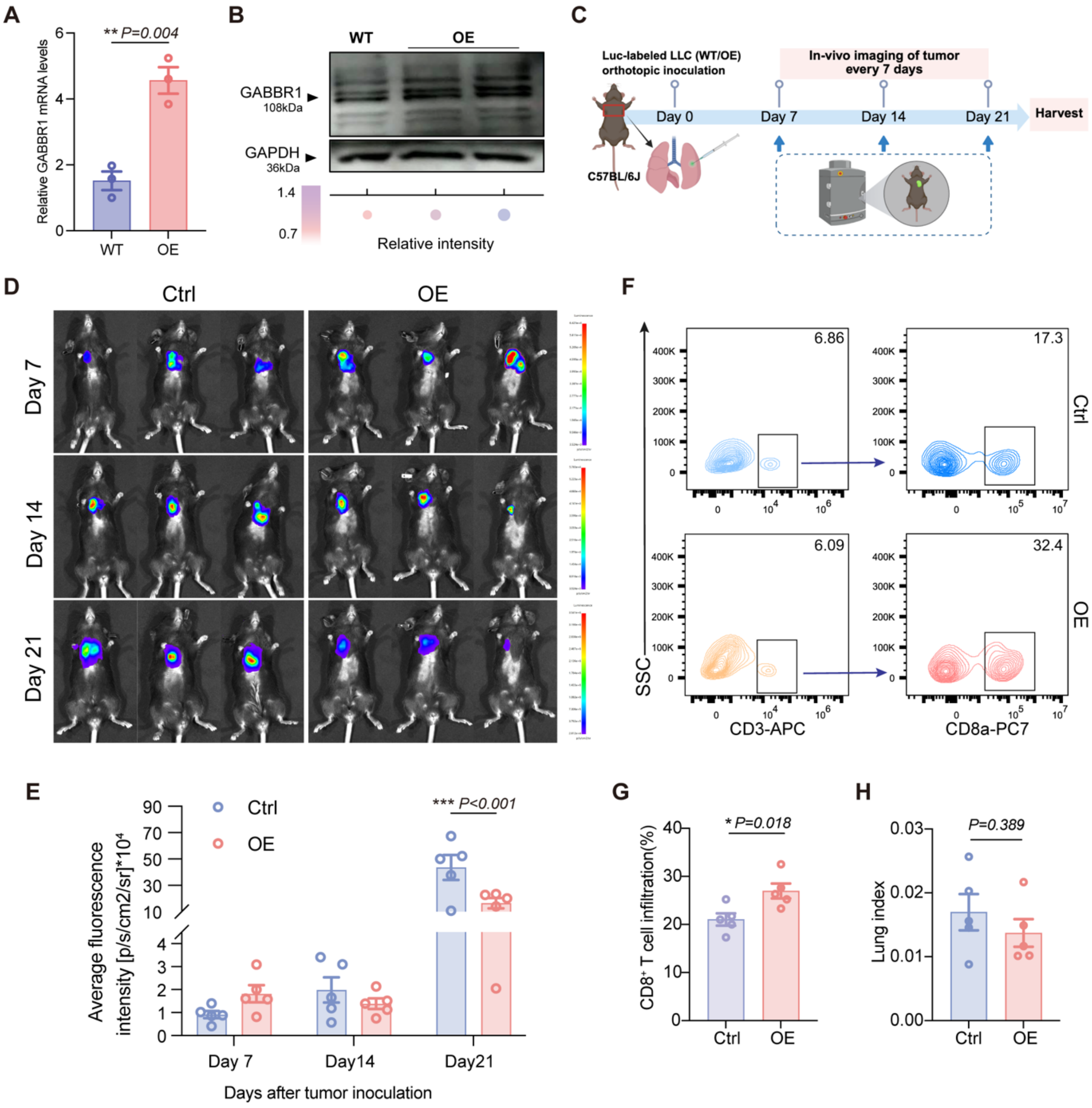

Figure 5 GABBR1 overexpression in tumor cells suppressed tumor growth with enhanced $CD8^+$ T cells infiltration *in vivo*. Establishment and validation of stable GABBR1-overexpressing (GABBR1-OE) LLC-Luc cells. GABBR1 overexpression was confirmed by RT-qPCR (A) and Western blot (B), compared to wild-type (WT) controls. C) Schematic diagram of orthotopically injecting LLC-Luc (WT) or LLC-Luc (GABBR1-OE) cells in C57BL/6J mice. Tumor size was monitored longitudinally using *in vivo* bioluminescence imaging every 7 days. All mice were sacrificed on day 22 for tumor collection and analysis (n=5 per group). (D) Representative *in vivo* bioluminescence images of lung tumor at different time points post-injection. E) Quantitative analysis of bioluminescent intensity. F) Flow cytometry plots showing the tumor infiltrating $CD3^+$ T cells and $CD8^+$ T cells isolated from lung tumors from experimental groups. G) Quantitative analysis of infiltrated $CD8^+$ T cells

from flow cytometric result. H) The lung index (lung weight/body weight) calculated at endpoint. Data are presented as mean ± SEM. Statistical significance was determined by two-way ANOVA (E) and two-tailed unpaired t-test (A, G, H).

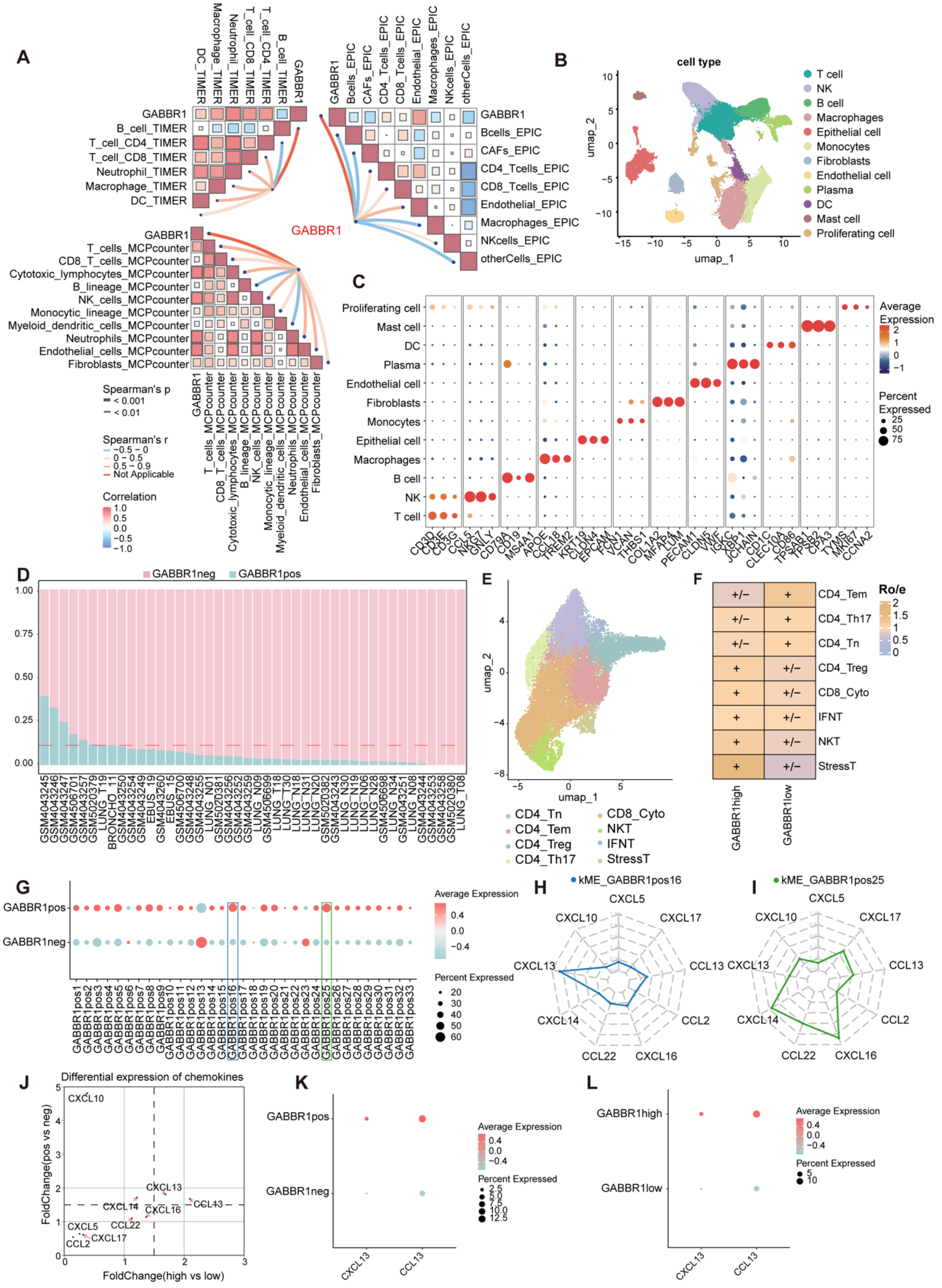

Figure 6 Comprehensive analysis of GABBR1 in bulk and single-cell RNA-seq datasets predict chemokines. A) The correlation between GABBR1 and the fraction of immune infiltration cells. B) A Uniform Manifold Approximation and Projection (UMAP) shows 112228 cells colored

by major cell types. C) Dot plots show the average expression of known markers in indicated clusters. Dot size represents the proportion of cells with expression in each cluster and color indicates expression intensity. D) The ratio of GABBR1-pos/neg epithelial cells in patients. E) UMAP plot of subtypes of T cells. F) Heatmap showing the result of the Ro/e. G) Comparison of module eigengene scores between GABBR1pos and GABBR1neg groups. H-I) The core chemokines within GABBR1pos16 (H) and GABBR1pos25 (I) module were identified and prioritized based on their kME values. J) Differential expression of chemokines. Scatter plot of fold changes in chemokine expression between GABBR1-high vs low (x-axis) and GABBR1-pos vs neg (y-axis). K-L) CXCL13 and CCL13 expression levels were compared between GABBR1-pos vs neg (K) and GABBR1-high vs low (L).

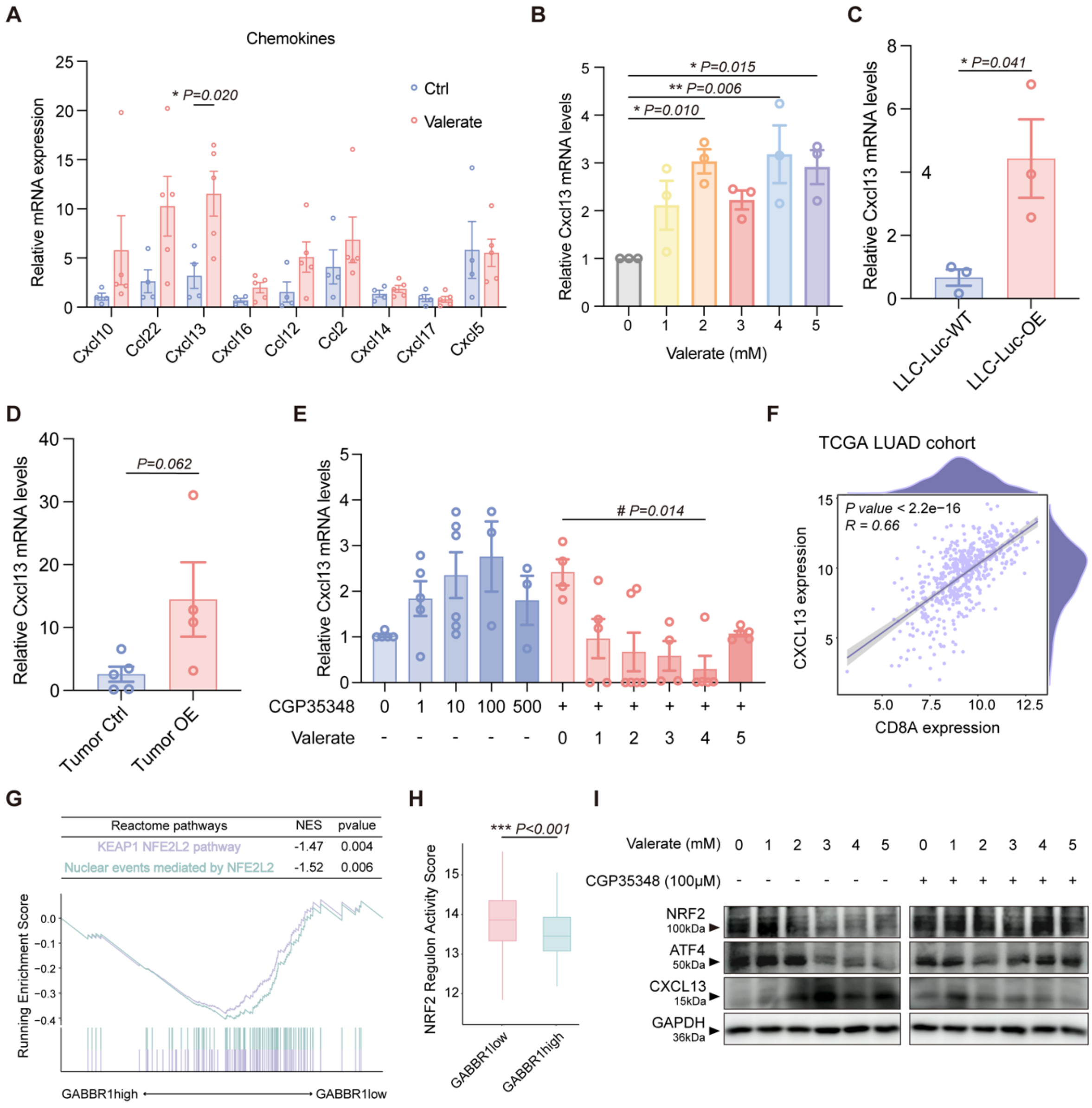

Figure 7 Valerate upregulates CXCL13 in the tumor microenvironment through GABBR1-ATF4-NRF2 axis. A) RT-qPCR analysis was conducted to measure the relative expression levels of the candidate chemokines in the tumors from valerate-treated mice and the controls (n=5 per group). B) RT-qPCR analysis of CXCL13 expression in LLC cells treated with elevated concentration of valerate (0, 1, 2, 3, 4, 5 mM) for 24 h. C, D) RT-qPCR analysis of CXCL13 expression in the wild type and GABBR1-OE LLC-Luc cells (C), and GABBR1-OE mice model (D) (n=5 per group). E) RT-qPCR analysis of CXCL13 in LLC cells treated with CGP35348 alone and co-treated with elevated concentration of valerate (0, 1, 2, 3, 4, 5 mM) and CGP35348 (100 μM) for 24h. F) Pearson correlation between CXCL13 and CD8A expression in tumors from patients in TCGA LUAD cohort. G) GSEA plot of significant Reactome pathways in comparison between the GABBR1high and GABBR1low groups in the TCGA LUAD cohort. H) Comparison of the regulon activity score of NRF2 between GABBR1

high and GABBR1 low groups in the TCGA LUAD cohort. I) Western blot analysis is performed to detect NRF2, ATF4 and CXCL13 protein levels in LLC cells treated with increasing concentration of valerate (0, 1, 2, 3, 4, 5 mM) with or without 100 μM of CGP35348. Data are presented as mean ± SEM. Statistical significance was determined by one way ANOVA (B, E), two-tailed unpaired t-test (A, C, D, H) and Pearson test (F).

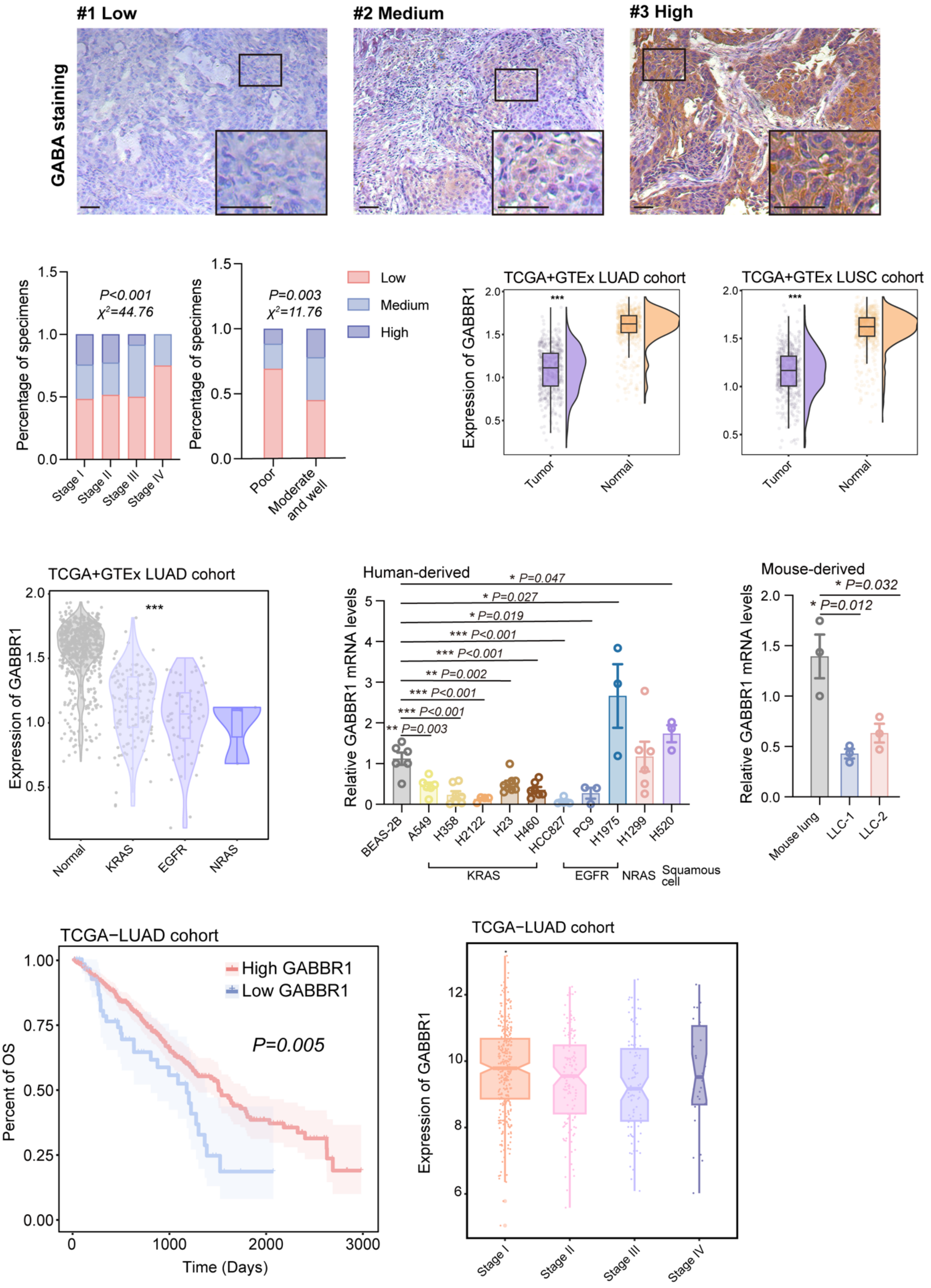

Figure 8 Clinical significance of GABBR1 as a promising therapeutic target to enhance anti-tumor immunity. A) Representative IHC images of GABA in clinical specimens from patients with lung cancer clarified as low, medium and high GABA (n=117). Scale bar =50 μm. B)

Quantification of GABA staining of samples from patients with different clinical stages and cell differentiation of lung cancer. C) Analysis of GABBR1 expression in tumors and normal tissues in TCGA LUAD, TCGA LUSC and GTEx cohort. D) Analysis of GABBR1 expression in tumors harboring different mutations (KRAS, EGFR, NRAS) in TCGA LUAD and GTEx cohort. E, F) RT-qPCR analysis of GABBR1 in lung adenocarcinoma cell lines and lung squamous cell lines. Human lung cancer cell lines harboring different mutations (KRAS, EGFR, NRAS). Mouse lung cancer cell line compared to the mouse lung tissues (F). G) Kaplan-Meier survival analysis correlating GABBR1 expression in the LUAD cohort with survival probability. H) Quantification of GABBR1 staining of samples from patients in TCGA LUAD cohort Data are presented as mean ± SEM. Statistical significance was determined by $\chi 2$ tests (B), two-tailed unpaired t-test (C), long-rank (G), one way ANOVA (D, E, F, H).

Supplementary Figures and Tables

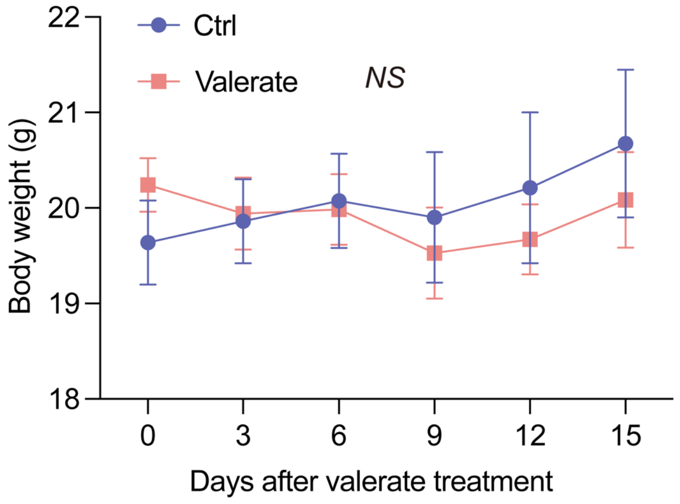

Figure S1 There was no significant change in body weight between valerate-treated group and the control group. Data are presented as mean ± SEM. Statistical significance was determined by two-way ANOVA.

A

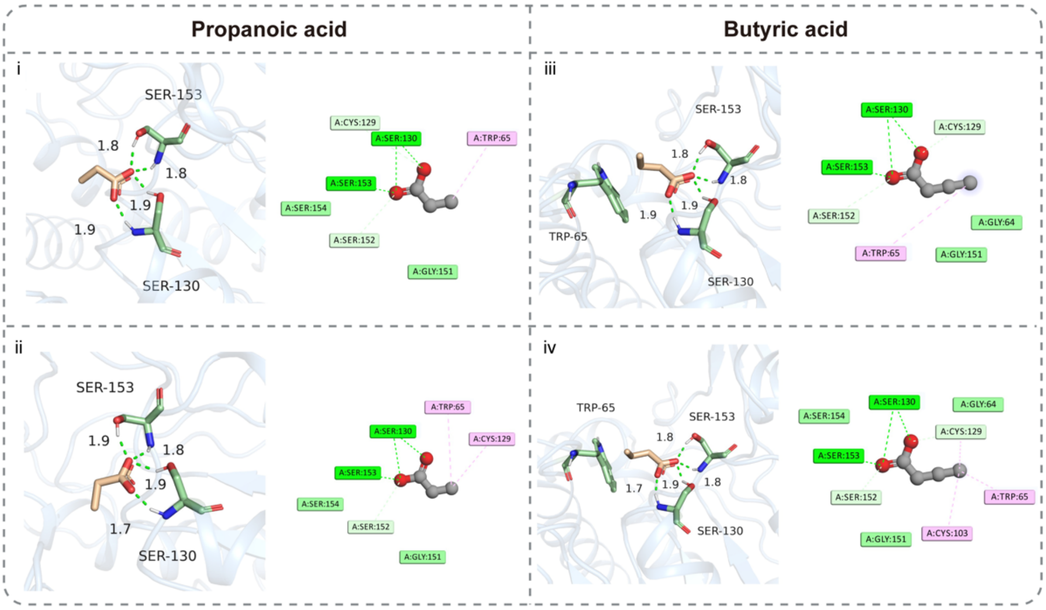

B

Compounds	SP Docking score (Kcal/mol)	XP Docking score (Kcal/mol)
Propanoic acid	-3.714	-2.616
Butyric acid	-4.697	-2.817

Carbon Hydrogen Bond
Ven der Waals
Conventional Hydrogen Bond
Alkyl

Figure S2 Comparative molecular docking of short-chain fatty acids to GABBR1. A) Three-dimensional (3D) and two-dimensional (2D) binding models of propanoic acid or butyric acid with the GABBR1 protein generated through standard-precision (SP) molecular docking (i, iii) and extra-precision (XP) docking (ii, iv). B) SP docking and XP docking score of compounds propanoic acid and butyric acid.

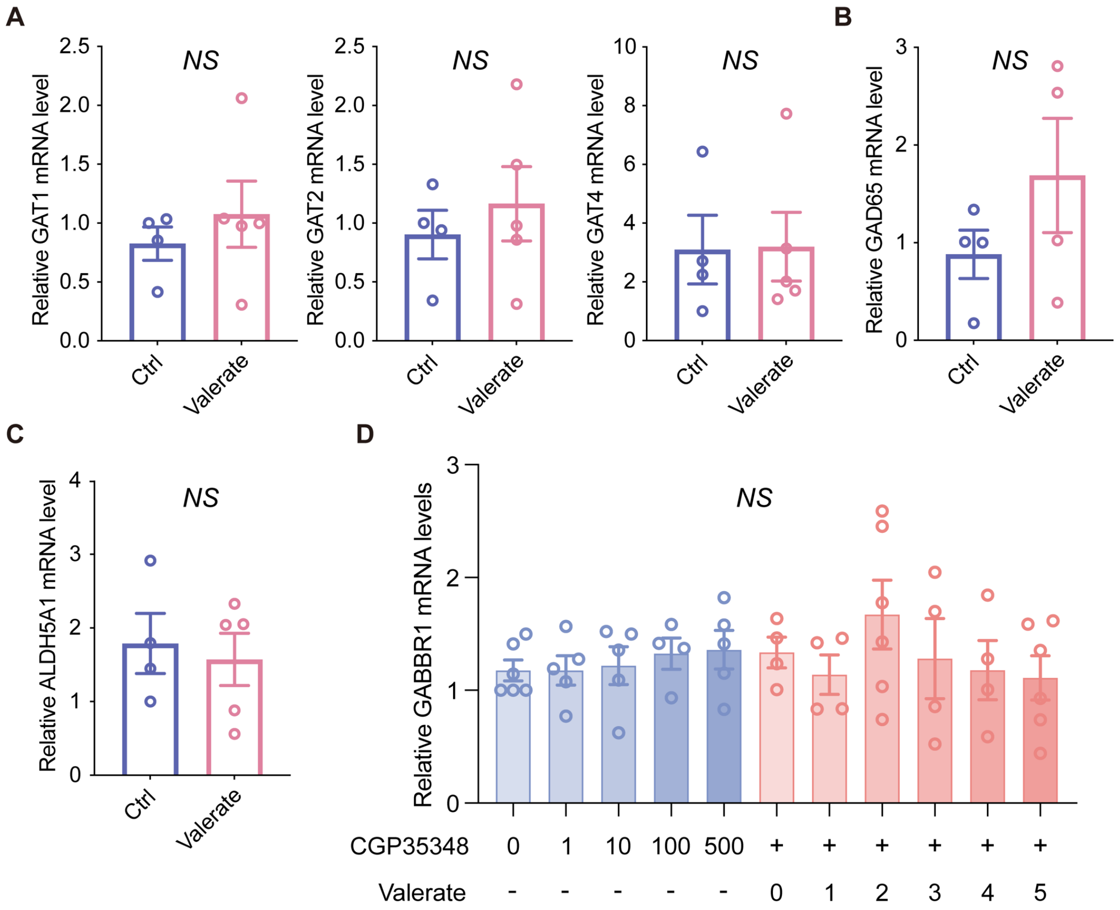

Figure S3 GABA metabolism was not altered by valerate; CGP35384 did not affect the expression level of GABBR1. A-C) RT-qPCR analysis of GAT1, GAT2, GAT4 (A), GAD65 (B), and ALDH5A1 (C) in tumor tissues from valerate-treated mice and the controls (n=4-5) D) RT-qPCR analysis of GABBR1 in LLC cells treated with CGP35384 alone, and increased concentration of valerate (0, 1, 2, 3, 4, 5 mM) combined with CGP35348 (100 μM) for 24 h. Data are presented as mean ± SEM. Statistical significance was determined by two-tailed unpaired t-test (A-C) and one-way ANOVA (D).

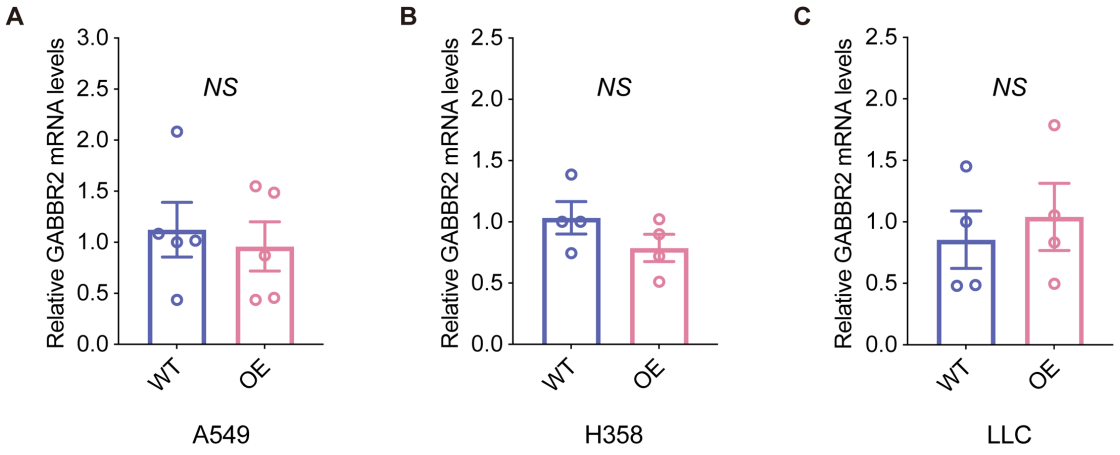

Figure S4 The GABBR2 expression was not significantly changed post-overexpression of GABBR1 in A549 (A), H358 (B) and LLC (C). Data are presented as mean ± SEM. Statistical significance was determined by two-tailed unpaired t-test (A-C).

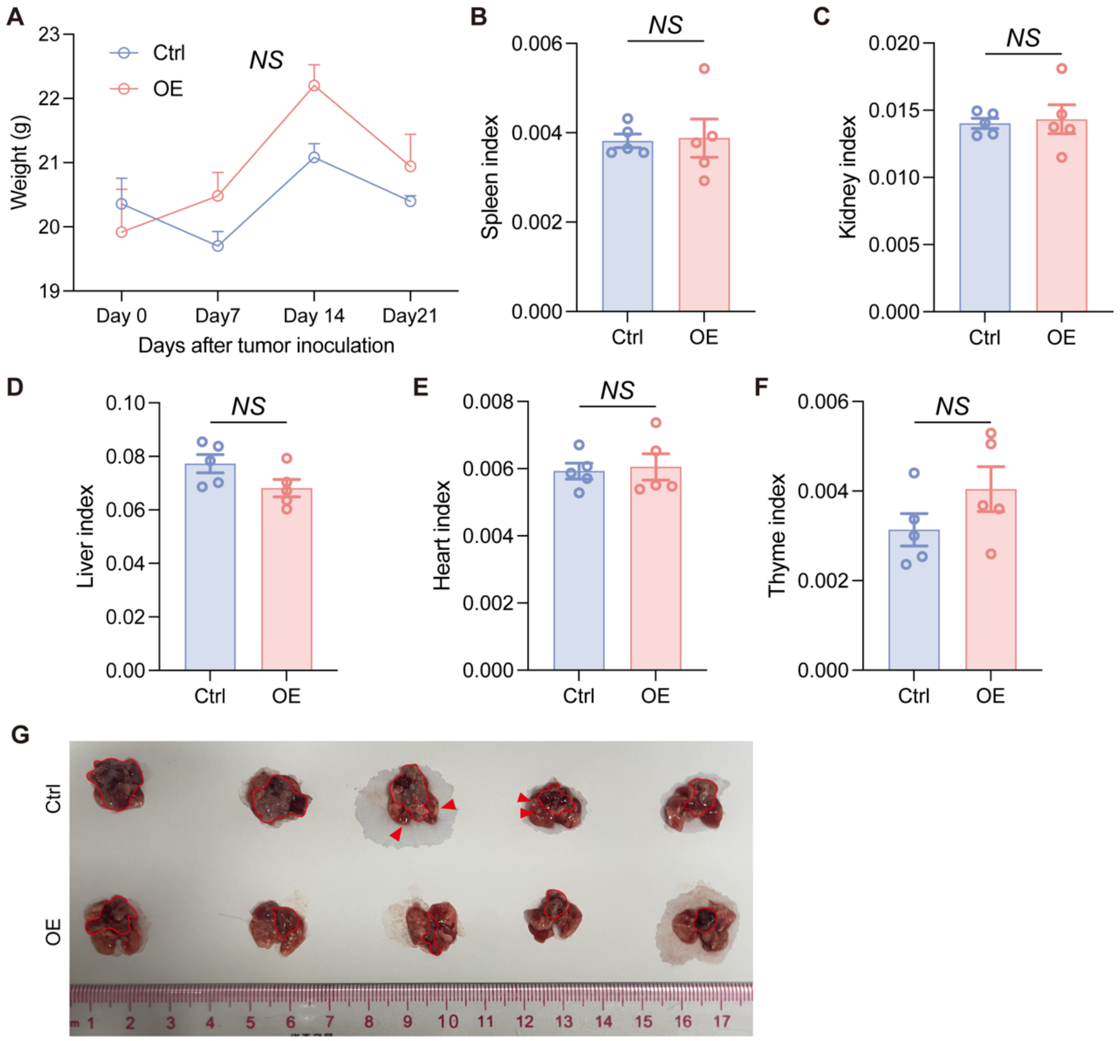

Figure S5 There was no change in body weight and organ index in GABBR1-OE group compared to the controls. A) The changed curve of body weight changes over 21 days post-inoculation between experimental groups. B-F) The changes of organ index (organ weight/body weight) at the endpoint between groups. G) Representative images of dissected lungs on day 22. The area outlined in red is the major tumor region; red arrow indicates representative nodules. Data are presented as mean ± SEM. Statistical significance was determined by two-way ANOVA (A) and two-tailed unpaired t-test (B-F).

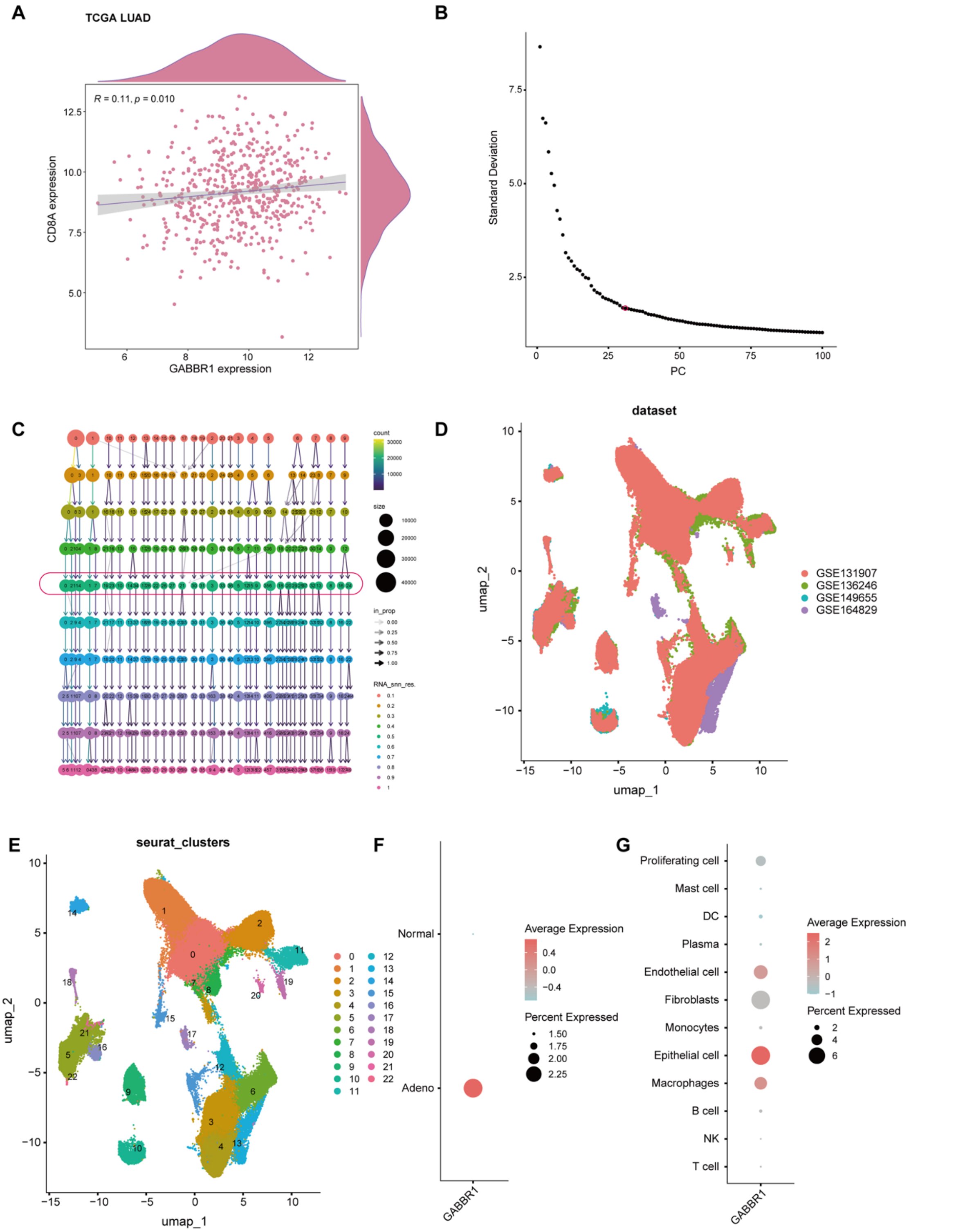

Figure S6 The expression level of GABBR1 in single cells cohorts. A) Correlation between GABBR1 and CD8A expression in the TCGA-LUAD bulk RNA-seq cohort. B) Elbow plot showing the standard deviation of each principal component (PC) from PCA. The inflection point (red dot) marks the selected number of principal components used for downstream analyses. C) Clustering hierarchy visualized using the clustree algorithm. D) UMAP visualization of four single cells cohorts. E) UMAP visualization of seurat clusters. F)

Comparison of the GABBR1 expression between adenocarcinoma and normal groups. G) The GABBR1 expression in the major cell types.

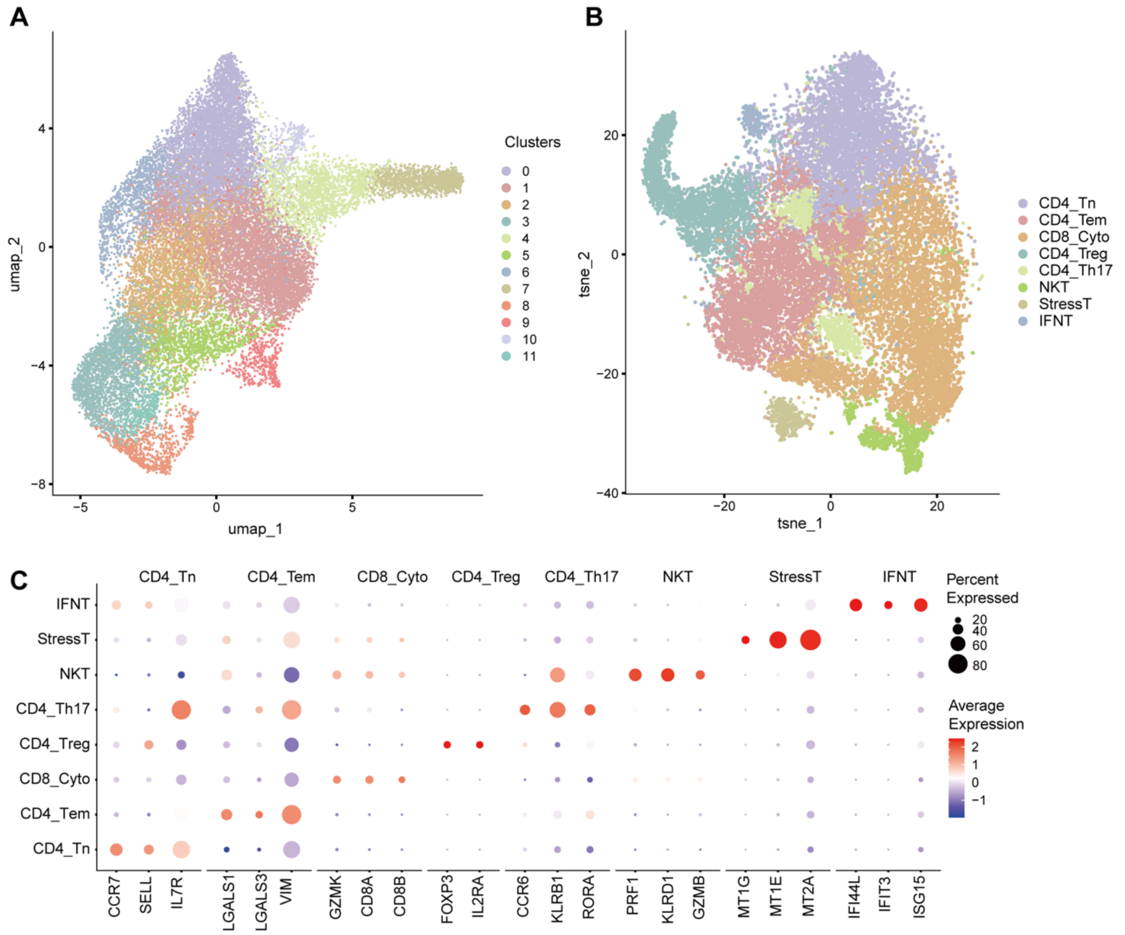

Figure S7 Identification and characterization of T cell subsets via unsupervised clustering and marker gene expression. A) UMAP visualization of T cells subclusters. B) UMAP visualization of T cells subtypes. C) Dot plots show the average expression of known markers in T cells subtypes.

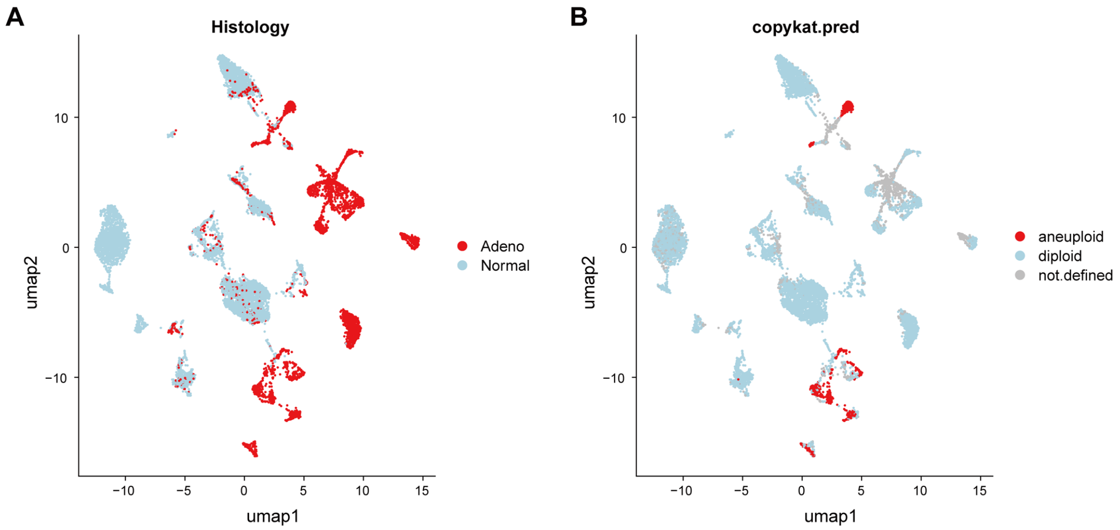

Figure S8 Identification of malignant epithelial cells in scRNA-seq cohorts. UMAP visualization of epithelial cells colored by histology-based annotation (A) and by CopyKAT-predicted groups (B).

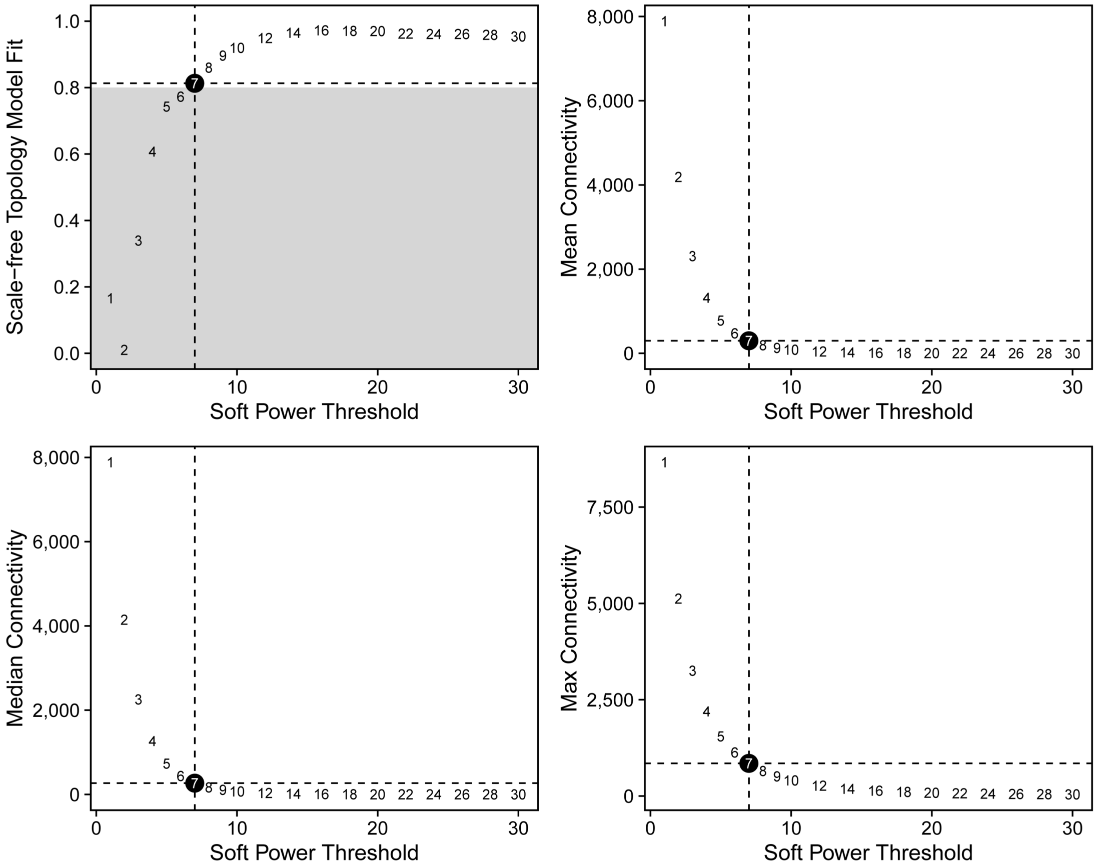

Figure S9 Evaluation of scale-free topology fit and mean connectivity for selection of the soft-thresholding power in WGCNA.

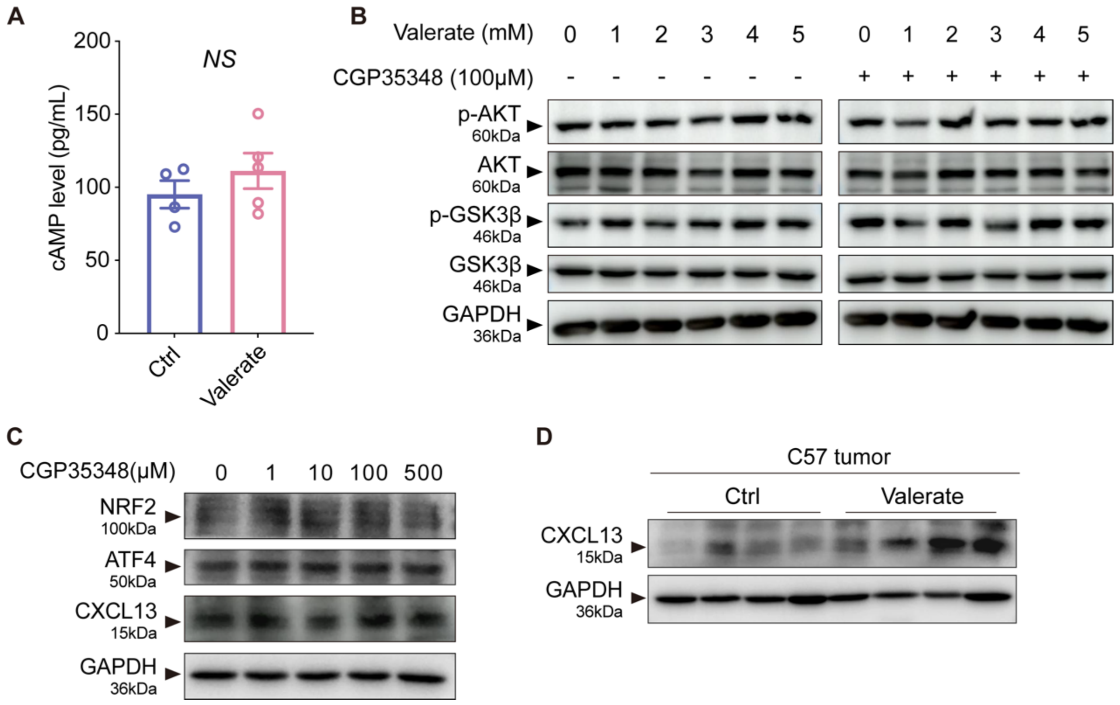

Figure S10 GABBR1-dependent signaling pathways in response to valerate and CGP35348. A) ELISA of cAMP level in tumor suspension from valerate-treated mice compared with controls. B) Western blot analysis detecting p-AKT and p-GSK3β protein levels in LLC cells treated with increasing concentration of valerate (0, 1, 2, 3, 4, 5 mM) with or without 100 μM of CGP35348. C) Representative Western blot of ATF4, NRF2 and CXCL13 in LLC cells treated with CGP35348 treatment (0, 1, 10, 100, 500 μM). D) Representative Western blot of CXCL13 in tumor tissues from mice treated with or without valerate. Data are presented as mean ± SEM. Statistical significance was determined by two-tailed unpaired t-test (A).

Table S1 Primers for qRT-PCR

Gene		Sequence
**Mouse**		
GABBR1	Forward	5′- TGGTTTCTCATCGGGTGGTAT -3′
	Reverse	5′- CCAAGGCCCAGATAGCATCA -3′
GABBR2	Forward	5′- GTTGTGCCTTTGAGGAGAGC -3′
	Reverse	5′- AGTCCACTCCGATGTAGCCT -3′
CXCL13	Forward	5′- TGAGGCTCAGCACAGCAA -3′
	Reverse	5′- ATGGGCTTCCAGAATACCG -3′
CCL22	Forward	5′- AAGCCTGGCGTTGTTTTGAT -3′
	Reverse	5′- CCTGGGATCGGCACAGATA -3′
CXCL10	Forward	5′- GGATGGCTGTCCTAGCTCTG -3′
	Reverse	5′- ATAACCCCTTGGGAAGATGG -3′
CXCL16	Forward	5′- TGTGGAACTGGTCATGGGAAG -3′
	Reverse	5′- AGCTTTTCCTTGGCTGGAGAG -3′
CCL12	Forward	5′- CCGGGAAGCTGTGATCTTCAGG -3′
	Reverse	5′- TGGGGAACTTCAGGGGGAAAT -3′
CCL2	Forward	5′- CAGATGCAGTTAACGCCCCA -3′
	Reverse	5′- TTGAGCTTGGTGACAAAAACTACAG -3′
CXCL14	Forward	5′- GAAGATGGTTATCGTCACCACC -3′
	Reverse	5′- CGTTCCAGGCATTGTACCACT -3′
CXCL17	Forward	5′- CCTCTCCCTTCCTTCTGTTGC -3′
	Reverse	5′- GCGACTTCCTGTGGTGCTTT -3′
CXCL5	Forward	5′- GGTCCACAGTGCCCTACG -3′
	Reverse	5′- GCGAGTGCATTCCGCTTA -3′
GAT1	Forward	5′- TGCTGCCGTGTGCATCGTGT -3′
	Reverse	5′- AGGGCCATTCTCAGGGCGCA -3′
GAT2	Forward	5′- CTTCTCCTTTGCCATCTGTCTGG -3′
	Reverse	5′- GCTGCTGTTGAGGATGCAAAGG -3′
GAT4	Forward	5′- CCTGAACAGTGCTACCAGCTTC -3′

	Reverse	5′- GGCATCATGGTGACAGCTTTGG -3′
GAD65	Forward	5′- AGCTGTGCGCTCTGCTCTATG -3′
	Reverse	5′- GCTCATTGGGGTAATGGAAATCG -3′
Aldh5a1	Forward	5′- CGCCTACGATGCCTTCAACA -3′
	Reverse	5′- AATACGGCGAGCTTCCTCTG -3′
β-actin	Forward	5′- CCACCATGTACCCAGGCATT -3′
	Reverse	5′- ACTCCTGCTTGCTGATCCAC -3′
**Human**		
GABBR1	Forward	5′- GCCACACTCCACAACCCTAC -3′
	Reverse	5′- ACTCTGGCGGAAAGTAATCTCAA -3′
GABBR2	Forward	5′- TGAAGGTGGGCGAGTACAAC -3′
	Reverse	5′- GCGGTTCGACACCTTGAAAC -3′
18S rRNA	Forward	5′- GCAATTATTCCCCATGAACG -3′
	Reverse	5′- GGCCTCACTAAACCATCCAA -3′

Table S2 Summary of fold changes and corresponding adjusted p values for all chemokines analyzed in the single-cell database (Figure 6J)

Chemokine	FC (pos vs. neg)	FC (high vs.low)	p.adj (pos vs. neg)	p.adj (high vs.low)
CXCL13	1.806	1.689	**0.003**	**0.042**
CCL13	1.677	2.096	0.228	**0.04**
CXCL16	1.136	1.367	**0.037**	**<0.001**
CCL22	1.105	1.131	0.087	0.395
CXCL14	1.719	1.217	**0.046**	0.072
CCL2	0.540	0.149	0.061	0.103
CXCL5	0.640	0.257	0.584	**0.004**
CXCL10	4.815	0.387	0.189	0.062
CXCL17	0.613	0.315	**0.001**	**<0.001**

Table S3 Clinical pathological characteristics correlation analysis with GABA expression in a 117 NSCLC cohort

Characteristics	Total	GABA expression			χ2	*P*-value
		**Low (%)**	**Medium (%)**	**High (%)**		
**Age (years)**					0.56	0.756
≤60	67	35 (52.2)	20 (29.9)	12 (17.9)		
>60	50	24 (48.0)	15 (30.0)	11 (22.0)		
**Gender**					0.005	0.998
Male	97	49 (50.5)	29 (29.9)	19 (19.6)		
Female	20	10 (50.0)	6 (30.0)	4 (20.0)		
**Smoking history**					2.363	0.307
Smoker	82	41 (50.0)	23 (28.0)	18 (22.0)		
Non-smoker	35	18 (51.4)	12 (34.3)	5 (14.3)		
**Pathological pattern**					1.906	0.386
Adenocarcinoma	63	29 (46.0)	21 (33.3)	13 (20.6)		
Squamous	54	30 (55.6)	14 (25.9)	10 (18.5)		
**Cell differentiation**					11.763	**0.003**
Poorly	26	18 (69.2)	5 (19.2)	3 (11.5)		
Moderately and well	91	41 (45.1)	30 (33.0)	20 (22.0)		
**TNM stage**					44.767	**< 0.001**
I	58	28 (48.3)	16 (27.6)	14 (24.1)		
II	31	16 (51.6)	8 (25.8)	7 (22.6)		
III	24	12 (50.0)	10 (41.7)	2 (8.3)		
IV	4	3 (75.0)	1 (25.0)	0 (0)		

Reference

1. Liang TL, Li RZ, Mai CT, Guan XX, Li JX, Wang XR, et al. A method establishment and comparison of in vivo lung cancer model development platforms for evaluation of tumour metabolism and pharmaceutical efficacy. Phytomedicine. 2022; 96: 153831.
2. Geng Y, Bush M, Mosyak L, Wang F, Fan QR. Structural mechanism of ligand activation in human GABA(B) receptor. Nature. 2013; 504: 254-9.
3. Shelley JC, Cholleti A, Frye LL, Greenwood JR, Timlin MR, Uchimaya M. Epik: a software program for pK(a) prediction and protonation state generation for drug-like molecules. J Comput Aided Mol Des. 2007; 21: 681-91.
4. Lua RC, Lichtarge O. PyETV: a PyMOL evolutionary trace viewer to analyze functional site predictions in protein complexes. Bioinformatics. 2010; 26: 2981-2.
5. Zeng D, Ye Z, Shen R, Yu G, Wu J, Xiong Y, et al. IOBR: Multi-Omics Immuno-Oncology Biological Research to Decode Tumor Microenvironment and Signatures. Front Immunol. 2021; 12: 687975.
6. Wu T, Hu E, Xu S, Chen M, Guo P, Dai Z, et al. clusterProfiler 4.0: A universal enrichment tool for interpreting omics data. Innovation (Camb). 2021; 2: 100141.
7. Zhang Y, Yang Z, Tang Y, Guo C, Lin D, Cheng L, et al. Hallmark guided identification and characterization of a novel immune-relevant signature for prognostication of recurrence in stage I–III lung adenocarcinoma. 2023; 10: 1657-74.
8. Gao R, Bai S, Henderson YC, Lin Y, Schalck A, Yan Y, et al. Delineating copy number and clonal substructure in human tumors from single-cell transcriptomes. Nat Biotechnol. 2021; 39: 599-608.
9. Morabito S, Miyoshi E, Michael N, Shahin S, Martini AC, Head E, et al. Single-nucleus chromatin accessibility and transcriptomic characterization of Alzheimer's disease. Nat Genet. 2021; 53: 1143-55.
